# Recent Publication Trends in Radiotherapy and Male Infertility over Two Decades: A Scientometric Analysis

**DOI:** 10.3389/fcell.2022.877079

**Published:** 2022-05-12

**Authors:** Shubhadeep Roychoudhury, Anandan Das, Manesh Kumar Panner Selvam, Saptaparna Chakraborty, Petr Slama, Suresh C. Sikka, Kavindra Kumar Kesari

**Affiliations:** ^1^ Department of Life Science and Bioinformatics, Assam University, Silchar, India; ^2^ Department of Urology, Tulane University School of Medicine, New Orleans, LA, United States; ^3^ Department of Animal Morphology, Physiology and Genetics, Faculty of Agrisciences, Mendel University in Brno, Brno, Czechia; ^4^ Department of Applied Physics, School of Sciences, Aalto University, Espoo, Finland

**Keywords:** radiotherapy, infertility, semen, reproductive hormones, omics, fertility preservation

## Abstract

Radiotherapy, a popular cancer management procedure, negatively impacts reproductive health particularly by reducing the fertility potential. The purpose of this study was to analyze the research trend in radiotherapy associated with male infertility over the past 20 years (2000-May 2021). SCOPUS database was used to retrieve relevant scientometric data (publication per year, affiliation, journals, countries, type of document and area of research) for different subgenres of radiotherapy and male infertility. A total of 275 articles were published related to radiotherapy and male infertility, with the United States being the most dominant country in research output in this field. Radiotherapy and male infertility research have shown positive growth over the last two decades. In-depth analysis revealed that publications (n) related to radiotherapy and male infertility research mainly focused its impact on semen parameters (n = 155) and fertility preservation techniques (n = 169). Our scientometric results highlight a limited research focus on the field of radiotherapy and its impact on male reproductive hormones. Furthermore, a significant lack of research was noticed in the area of omics and male reproductive organs linked to radiotherapy. Substantial research is warranted to further decipher the effect of radiotherapy, at molecular level, leading to male infertility.

## Introduction

Infertility is described as the incompetence of couples to conceive after at least a year of regular unprotected intercourse (World Health Organization, 2020a). Currently, it has been estimated that 8–12% of couples worldwide are infertile with male factor being the primary cause in approximately 50% of those couples (Agarwal et al., 2020). Male fertility is often influenced by environmental and/or occupational factors such as pesticides (Lwin et al., 2018), bisphenol A (BPA)-based polycarbonate plastics (Xu et al., 2005) and heavy metals, which tend to disrupt the functions of the hypothalamic-pituitary-gonadal (HPG) axis (Roychoudhury et al., 2021). In addition, certain lifestyle practices, such as cigarette smoking, alcohol consumption, illicit drug use, obesity, psychological stress, advanced parental age, dietary practices, and heavy caffeine consumption, are known to affect semen quality (Sharma et al., 2013; Durairajanayagam, 2018; Roychoudhury et al., 2021). Semen analysis, which is considered the cornerstone for laboratory evaluation of male infertility, along with a detailed medical and sexual history as well as a physical examination can provide precise information on the fertility potential of a man (Barrat, 2007; Sunder and Leslie, 2021). Several studies have reported a decline in sperm quality, such as semen volume, sperm concentration, count, motility, and morphology, in cancer patients receiving radiotherapy and chemotherapy (Gandini et al., 2006; Caponecchia et al., 2016; Xu et al., 2019), which may persist for several years or may even be permanent (Meistrich, 2013).

International Agency for Research on Cancer (IARC) has reported 19.3 million new cancer cases in the year 2020 and about 10 million of them succumbed to death (Sung et al., 2021; World Health Organization, 2020b). An increasing trend was observed in the number of cancer patients relying on the modern therapeutic measures such as radiotherapy for its treatment and management (Miller et al., 2019). Nearly 50% of all cancer patients are subjected to radiotherapy at least once in the course of their disease (Baskar et al., 2012). Frequent use of radiotherapy increases the likelihood of fertility disorders in both men and women (Biedka et al., 2016). Radiotherapy negatively impacts the spermatogenesis process which may damage spermatocytes and spermatids (Ogilvy-Stuart and Shalet, 1993). Furthermore, radiotherapy can disrupt the proliferative capacity of Leydig cells and Sertoli cells, thereby leading to hormonal imbalance and sperm abnormalities (Tsatsoulis et al., 1990). Exposure to low radiation doses reportedly decreases semen volume and leads to temporary oligozoospermia (Mazur-Roszak et al., 2005). Moreover, radiation may induce reproductive toxicity in males (Fukunaga et al., 2022) and can generate mitochondrial ROS in mammalian spermatozoa (Aitken et al., 2022). Radiotherapy is also being used in the management of testicular cancer and prostate cancer. Nearly a 30% of decline in fertility potential was reported in testicular cancer patients undergoing radiotherapy (Huyghe et al., 2004). In addition, radiotherapy directed to the brain negatively impacts the functions of hypothalamus and pituitary gland that may disrupt the production of luteinizing hormone (LH) and follicle-stimulating hormone (FSH) (Pekic et al., 2000). All these research findings have led radiotherapy-induced infertility to become one of the most important issues in modern medicine (Biedka et al., 2016).

Scientometrics is an emerging field in science which deals with quantitative analysis of published scholarly literature that aims to focus on the expansion of a specific field of study through the assessment of bibliometric data (Baskaran et al., 2019; Agarwal et al., 2022). Previous bibliometric and scientometric reports indicated a considerable growth in male infertility research (Baskaran et al., 2019; Makkizadeh and Bigdeloo, 2019; Agarwal et al., 2021; Baskaran et al., 2021). Similarly, other bibliometric studies quantitatively analyzed global radiotherapy research and intraoperative radiotherapy (Sole et al., 2014; Aggarwal et al., 2018). With steady increase in cancer cases, radiotherapy has become one of the most reliable therapeutic interventions across the world. Radiotherapy-induced infertility has become a major concern among the male cancer patients. Hence, it is important to understand the research trends in the area of radiotherapy and male infertility. The main objective of this study is to conduct an in-depth scientometric analysis of publications related to various aspects of radiotherapy and male infertility over the past two decades.

## Materials and Methods

### Ethics Statement

This study did not involve the participation of human or animal subjects and was conducted using the scientometric data retrieved from the SCOPUS database. It is therefore considered to be excluded from review by the Institutional Review Board.

### Data Source and Retrieval Strategy

The present scientometric analysis was conducted using the SCOPUS database. It covers more than 84 million documents, more than 1.8 billion cited references dating back to 1970, and 17.5 million author profiles (Elsevier, 2021).

**FIGURE 1:**
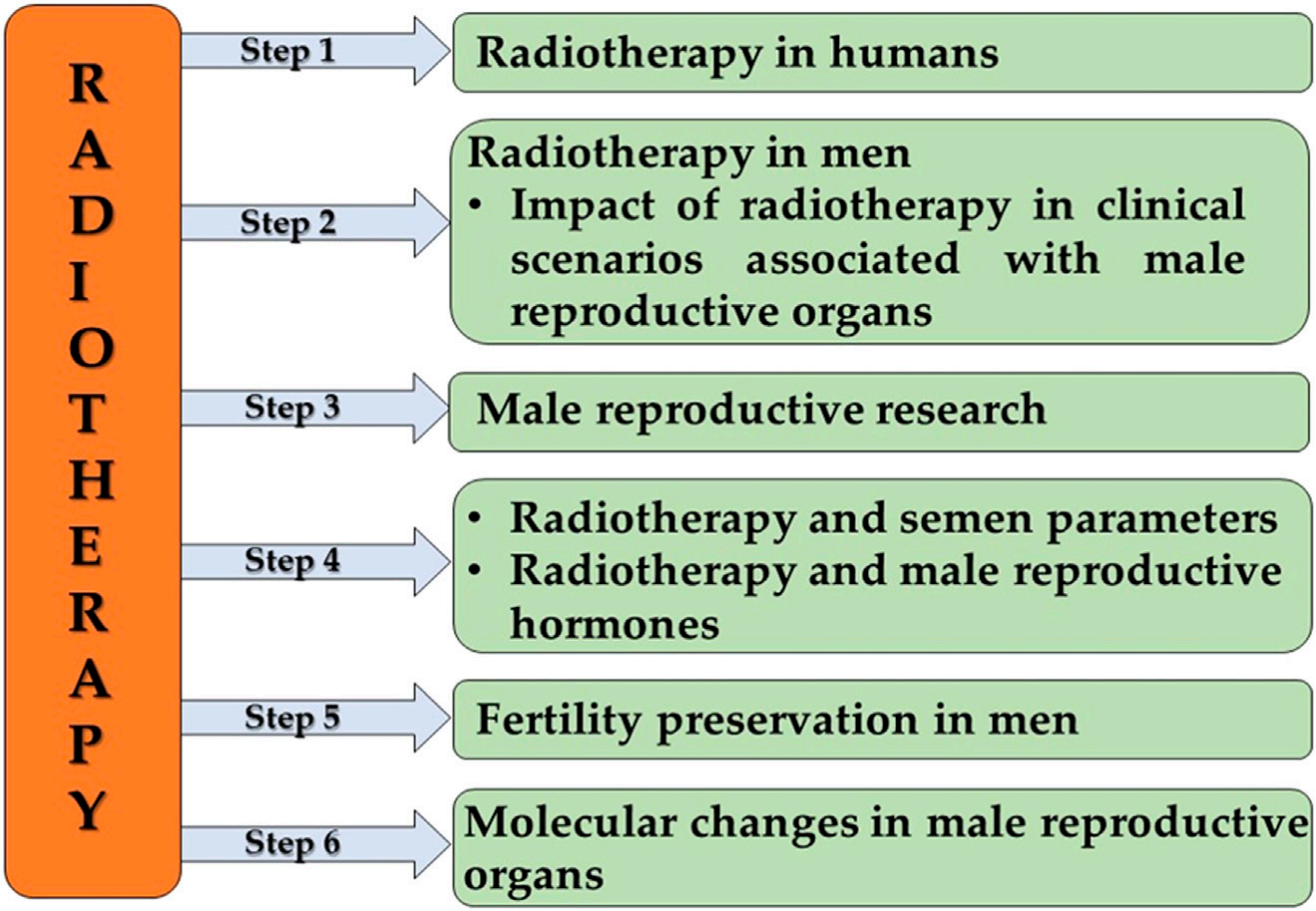
The framework of scientometric analysis. Step 1 analyzed the publication trend in radiotherapy in humans, followed by step 2 which included the analysis of publication trend in radiotherapy in men, focusing on the impact of radiotherapy in clinical scenarios associated with male reproductive organs. Step 3 analysis was focused on publication trend in radiotherapy and male reproductive research. Step 4 analyzed two aspects, publication trends associated with the impact of radiotherapy on semen parameters and male reproductive hormones. Steps 5 and 6 analyzed the publication trends in radiotherapy associated with fertility preservation in men and molecular changes in male reproductive organs, respectively.

Literature search was limited to human studies that were published from 2000 to May 2021. Search was carried out in six steps (Supplementary Table S1) as illustrated in Figure 1. Relevant keywords were selected for each step, and in some cases, an “asterisk” (*) was used after the keyword so that the search could include all the variants of that particular word (Supplementary Table S1). We also used multiple Boolean operators like “AND”, “OR”, “NOT” and “AND NOT” to obtain results relevant to our study and excluded the irrelevant publications. Apart from that, functions like “TITLE-ABSTRACT” and “TITLE-ABSTRACT-KEYWORDS” were used to extract maximum number of relevant articles. List of articles for each step was extracted and were subjected to screening of title and abstract. The relevance of all the articles were evaluated independently by two investigators (AD, SC) and validated by three experienced researchers (SR, MKPS, KKK) in the area of male infertility. Animal studies were excluded, and criteria for excluding other articles from analysis were listed in Supplementary Table S2.

### Scientometric Analysis

The scientometric data for the number of documents based on the year of publication, journal, country, affiliation, document type, and subject area were retrieved from the SCOPUS database. These extracted scientometric data were saved as comma-separated value (CSV) files and were then, for the sake of detailed analysis, converted to Microsoft Excel files.

The geographic mapping of publication trends on radiotherapy and male infertility research across the world was done using Tableau Desktop (Tableau, 2021). The network map on international collaborations was generated using the VOS viewer software (van Eck and Waltman, 2011). The geographical and network mapping aims to reveal international collaborative networks and macro scale multilevel structure of the concerned research area (Elsevier, 2021).

Additionally, we have calculated the citation rate of the articles addressing the impact of radiotherapy on semen parameters, reproductive hormones, and fertility preservation. Scientific impact of the articles is influenced by both citation number and publication year. To nullify this influence, we have made use of the citation rate (Ellul et al., 2017; Bullock et al., 2018) by applying the following formula:

$$\text{Citation}\;\text{rate}=\frac{\text{number}\;\text{of}\;\text{citations}}{2021-\text{year}\;\text{of}\;\text{publication}}$$

## Results

### Publication Trend in Radiotherapy Research

**FIGURE 2:**
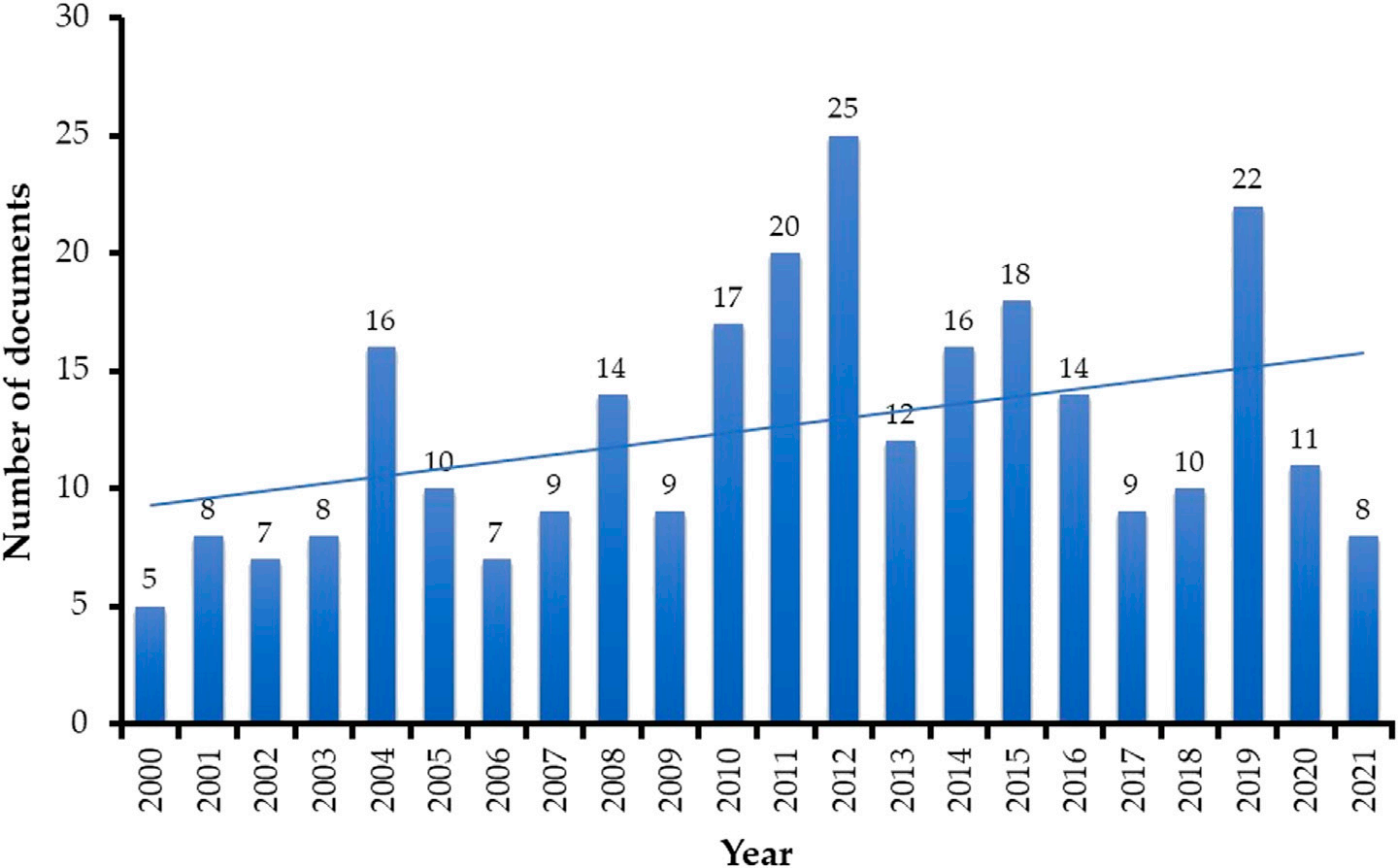
Number of publications per year over the past two decades (2000 to May 2021) related to the impact of radiotherapy research on male fertility. The trendline signifies an overall steady increase in research focus on this field throughout the last 20 years.

**FIGURE 3:**
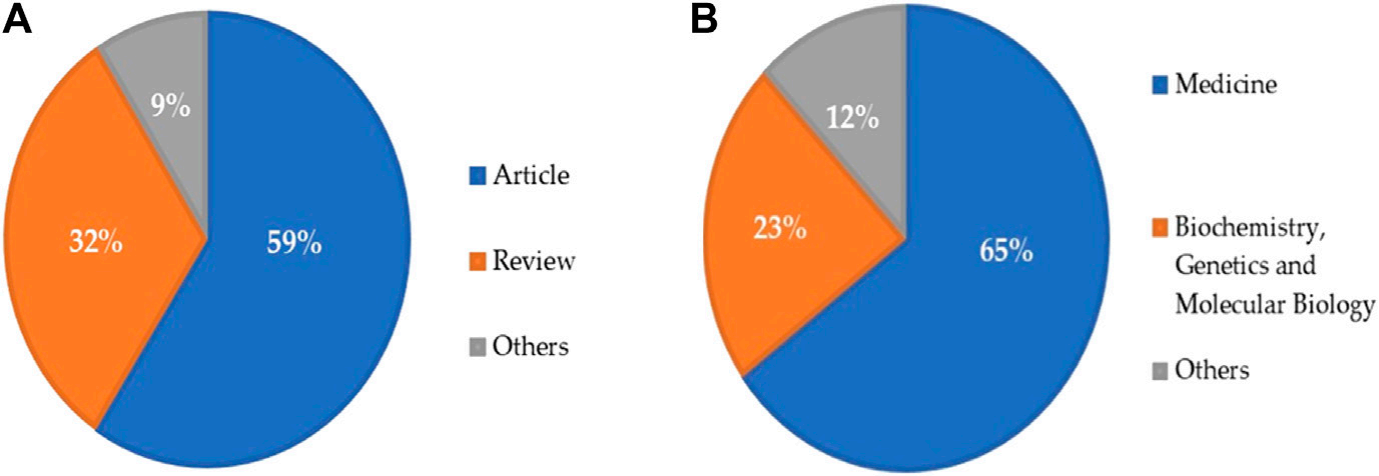
Research trend based on the **(A)** type of documents and **(B)** subject area published during the past two decades (2000 to May 2021) related to radiotherapy research in male infertility.

**FIGURE 4:**
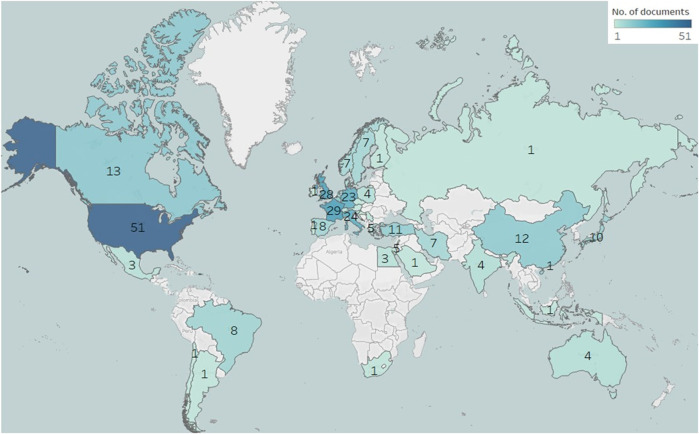
Geomap showing the distribution of publications from countries contributing to the field of research concerning radiotherapy and its impact on male fertility.

Scientometric analysis revealed that a total of 744 articles related to radiotherapy research in humans were published in the last 20 years (2000—May 2021), averaging around 34 publications per year. Whereas, 275 articles were published in the field of radiotherapy research in male infertility (*R* ^2^ = 0.1381) (Figure 2). Most of the publications (n) were original articles (n = 163, 59%) and review articles (n = 87, 32%) (Figure 3A). The research was mainly focused in the areas of Medicine (n = 252, 65%) and Biochemistry, Genetics and Molecular Biology (n = 88, 23%) (Figure 3B). Countries and top five institutes involved in male fertility and radiotherapy research are presented in Figure 4 and Table 1, respectively.

**TABLE 1:** Top five institutions contributing to the field of research related to radiotherapy and its impact on male fertility.

Institutions (Rank)	Number of Publications	Percentage of Total Publications (%)
Sapienza Università di Roma, Italy (1)	8	2.29
The University of Edinburgh, United Kingdom (2)	7	2.00
Memorial Sloan-Kettering Cancer Center,United States (3)	6	1.71
University of Texas MD AndersonCancer Center, United States (3)	6	1.71
Rikshospitalet-Radiumhospitalet HF, Norway (3)	6	1.71

An in-depth analysis revealed that only 25.45% (70/275) articles discussed the use of radiotherapy in different clinical scenarios associated with male reproductive organs. Among these, 62.86% (n = 44) and 35.71% (n = 25) publications were original and review articles, respectively.

### Publication Trend in Radiotherapy and Male Reproductive Research

**FIGURE 5:**
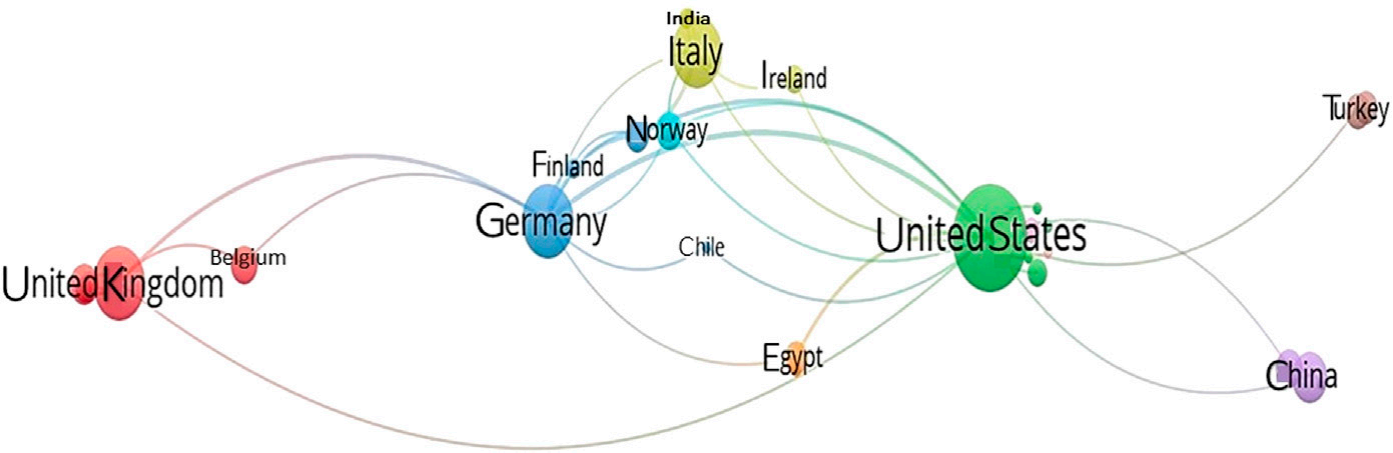
Network map showing the international collaboration based on publications in the field of radiotherapy and male reproductive research.

**FIGURE 6:**
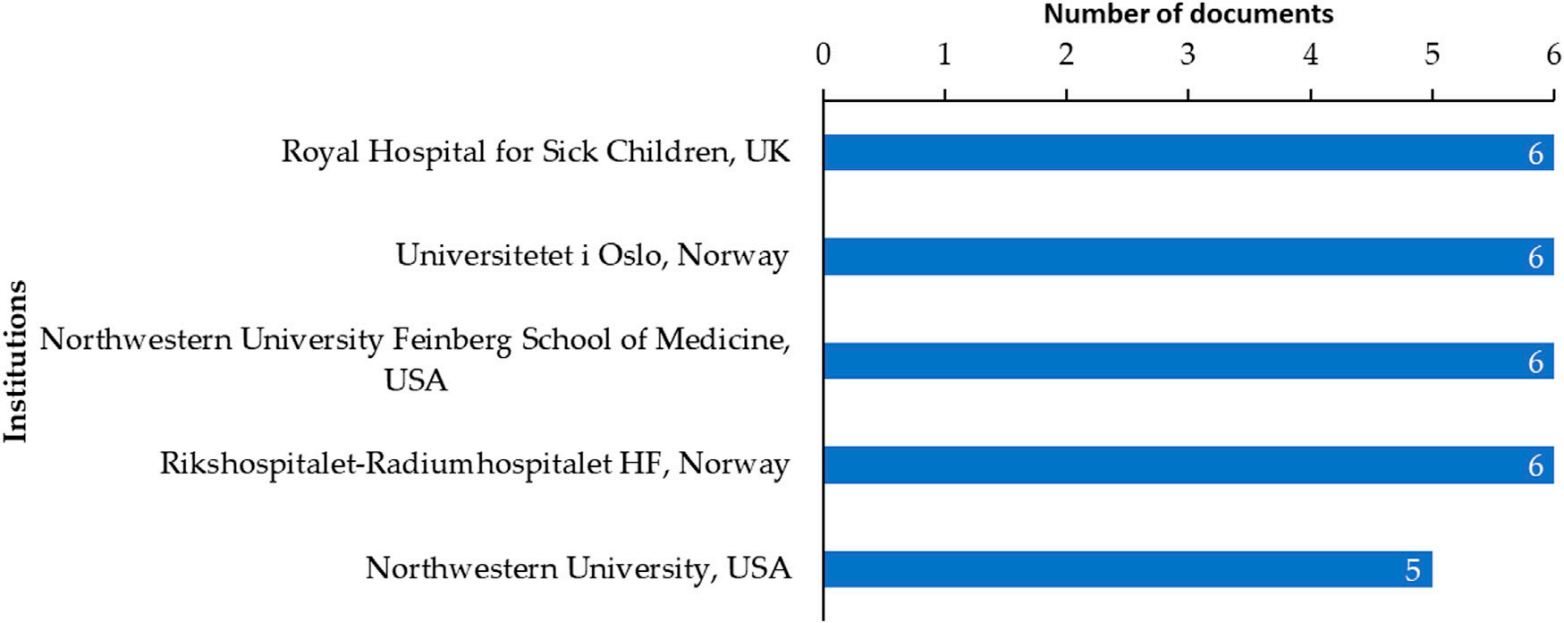
Top 5 institutions contributing to the field of radiotherapy and male reproductive research.

Research trend analysis revealed that 233 articles were published during the period from the year 2000 to May 2021 on radiotherapy and male reproductive research. Most of the publications were original articles (n = 116, 49.79%) and review articles (n = 91, 39.06%) mainly focused on the areas of Medicine (n = 215, 67.40%) and Biochemistry, Genetics and Molecular Biology (n = 63, 19.75%). Collaboration between the countries is presented as a network map (Figure 5) and top five institutes contributing towards this field of research are presented in Figure 6. Fertility and Sterility (n = 9), Human Reproduction (n = 6), and Andrologie (n = 4) were the top three journals publishing in this area.

### Publication Trend in Impact of Radiotherapy on Semen Parameters and Male Reproductive Hormones

Publication trend analysis on the impact of radiotherapy on semen and sperm parameters resulted in a total of 155 relevant articles published between 2000 and May 2021. Majority of the publications were original articles (n = 94, 60.64%) and reviews (n = 41, 26.45%). List of articles categorized based on semen parameters, such as azoospermia, oligozoospermia, and teratozoospermia, are included in Supplementary Table S3. Most of the publications were original articles (n = 20, 68.96%) followed by review papers (n = 7, 24.13%). There is a very limited number of publications which affirm that radiotherapy increases LH (n = 3) and FSH (n = 5) levels and decreases the GnRH (n = 2) level, which calls for further research to make a conclusive statement regarding this aspect. Studies reporting the reduction in testosterone, LH, FSH, and gonadotropin-releasing hormone (GnRH) are included in Supplementary Table S3. Top five countries and institutes contributing towards this field of research are presented in Table 2. Highly cited top 10 articles related to semen parameters and reproductive hormones are presented in Table 3.

**TABLE 2:** Top five countries and institutions contributing in the field of radiotherapy associated with semen parameters and male reproductive hormones.

	Semen Parameters (n = 155)	Reproductive Hormones (n = 29)
Countries	United States (n = 31)	France (n = 4)
	Germany (n = 20)	Germany (n = 4)
	United Kingdom (n = 19)	United Kingdom (n = 4)
	France (n = 18)	Belgium (n = 2)
	Italy (n = 12)	Canada (n = 2)
Institutions	Catholic University of Louvain, Belgium (n = 6)	Catholic University of Louvain, Belgium (n = 2)
	Cleveland Clinic Foundation, United States (n = 6)	Université McGill, Canada (n = 2)
	Royal Hospital for Sick Children, United Kingdom (n = 6)	Universitetet i Oslo, Norway (n = 2)
	Hospital Charles Nicolle, Tunisia (n = 6)	King’s College London, United Kingdom (n = 2)
	Charité—Universitätsmedizin Berlin, Germany (n = 6)	Cliniques Universitaires Saint-Luc, Belgium (n = 2)

**TABLE 3:** Top 10 articles investigating radiotherapy and male fertility each from the aspect of semen parameters, reproductive hormones and fertility preservation based on the citation rate.

Aspect	Rank	Manuscript (First Author, Title and year)	Total Citation	Citation Rate
Semen parameters	1	Aloisio GM. PAX7 expression defines germline stem cells in the adult testis, 2014	104	14.8
	2	Dohle GR. Male infertility in cancer patients: Review of the literature, 2010	161	14.6
	3	Wallace WHB. Oncofertility and preservation of reproductive capacity in children and young adults, 2011	128	12.8
	4	Paoli D. Spermatogenesis in Hodgkin’s lymphoma patients: A retrospective study of semen quality before and after different chemotherapy regimens, 2016	33	6.6
	5	Ding J. Protection of murine spermatogenesis against ionizing radiation-induced testicular injury by a green tea polyphenol, 2015	37	6.2
	6	Thomson AB. Late reproductive sequelae following treatment of childhood cancer and options for fertility preservation, 2002	113	5.94
	7	Van Casteren NJ. Gonadal dysfunction in male cancer patients before cytotoxic treatment, 2010	65	5.9
	8	Brougham MFH. Subfertility in children and young people treated for solid and haematological malignancies, 2005	91	5.7
	9	Maggelsen H. The effects of cancer and cancer treatments on male reproductive function, 2006	78	5.2
	10	Brusamolino E. Treatment of early-stage Hodgkin’s disease with four cycles of ABVD followed by adjuvant radiotherapy: Analysis of efficacy and long-term toxicity, 2000	63	3.0
Reproductive hormones	1	Brydoy M. Paternity following treatment for testicular cancer, 2005	181	11.3
	2	Van Casteren NJ. Gonadal dysfunction in male cancer patients before cytotoxic treatment, 2010	65	5.9
	3	De Felice F. Radiation effects on male fertility, 2019	9	4.5
	4	Trabado S. Male acquired hypogonadotropic hypogonadism: Diagnosis and treatment, 2012	29	3.2
	5	Brydoy M. Sperm counts and endocrinological markers of spermatogenesis in long-term survivors of testicular cancer, 2012	27	3.0
	6	Bojanic N. Testis sparing surgery in the treatment of bilateral testicular germ cell tumors and solitary testicle tumors: A single institution experience, 2015	17	2.8
	7	Ebert AK. Genital and Reproductive Function in Males After Functional Reconstruction of the Exstrophy-Epispadias Complex-Long-Term Result, 2008	29	2.2
	8	Meistrich ML. Suppression of testosterone stimulates recovery of spermatogenesis after cancer treatment, 2003	33	1.8
	9	Pfitzer C. Dynamics of fertility impairment in childhood brain tumour survivors, 2014	8	1.1
	10	Pasqualotto FF. Detection of testicular cancer in men presenting within fertility, 2003	12	0.6
Fertility preservation	1	Dohle GR. Male infertility in cancer patients: Review of the literature, 2010	161	14.6
	1	Onofre J. Cryopreservation of testicular tissue or testicular cell suspensions: A pivotal step in fertility preservation, 2016	73	14.6
	2	Fallat ME. Preservation of fertility in pediatric and adolescent patients with cancer, 2008	160	12.3
	3	Brydoy M. Paternity following treatment for testicular cancer, 2005	181	11.3
	4	Jahnukainen K. Testicular function and fertility preservation in male cancer patients, 2011	99	9.9
	5	Vassilakopoulou M. Anticancer treatment and fertility: Effect of therapeutic modalities on reproductive system and functions, 2016	37	7.4
	6	Kort JD. Fertility issues in cancer survivorship, 2014	45	6.4
	7	Meseguer M. Sperm cryopreservation in oncological patients: A 14-years follow-up study, 2006	93	6.2
	8	Thomson AB. Late reproductive sequelae following treatment of childhood cancer and options for fertility preservation, 2002	113	5.9
	9	Paoli D. Sperm cryopreservation: Effects on chromatin structure, 2014	31	4.4

### Publication Trend in the Impact of Radiotherapy and Fertility Preservation in Men

A total of 169 articles were published in the last 20 years that discussed radiotherapy and fertility preservation in men. Majority of the articles belonged to the area of medicine (n = 163, 73.09%) which was followed by Biochemistry, Genetics and Molecular Biology (n = 42, 18.83%). Fertility and Sterility (n = 10, 5.91%) and Human Reproduction (n = 8, 4.73%) were the top journals publishing in this area followed by Reproductive Biomedicine Online (n = 4, 2.37%). The United States (n = 33, 19.52%) was the leading country which published scientific literature in this field. In-depth analysis revealed that fertility preservation techniques such as vitrification (n = 5), cryopreservation of testicular tissue (n = 100), and sperm (n = 37) are impacted by radiotherapy. Top 10 highly cited articles addressing the impact of radiotherapy on fertility preservation in men were included in Table 3.

### Publication Trend in Impact of Radiotherapy and Molecular Changes in Male Reproductive Organs

Finally, we analyzed publication trend on impact of radiotherapy on molecular changes in male reproductive organs. Only two articles were published in the last 20 years. Out of the two publications, one is an original article (n = 1), and the other one is a review (n = 1).

## Discussion

Radiotherapy-induced male infertility is one of the important reproductive health concerns among cancer survivors (Vakalopoulos et al., 2015; Biedka et al., 2016). In the current study, scientometric approach was used to investigate the publication trends associated with radiotherapy and male infertility research during the last two decades. Scientometric analysis provides insights about the influence of scholarly research articles on the direction and progress of a specific field of study (Cooper, 2015; Roldan-Valadez et al., 2019). SCOPUS, one of the largest curated abstract and citation databases, was used in this study. It is an ideal literature retrieval platform for scientometric studies (Baas et al., 2020). Recently, several investigators also used similar methodology in their scientometric studies and analyzed the distribution of publications based on the specific subtopics (Bernabo et al., 2017; Baskaran et al., 2019; Martynov et al., 2020; Baskaran et al., 2021).

Cancer is a leading cause of mortality and morbidity around the world, accounting for nearly 10 million deaths in 2020 (Sung et al., 2021; World Health Organization, 2020b). Traditional methods such as radiotherapy has been reported as one of the most popular nonsurgical treatment options for malignant tumours (Thun et al., 2010; Mohan et al., 2019). It is cost-efficient and accounts for only about 5% of the total cost of cancer care (Ringborg et al., 2003). However, radiotherapy is strongly associated with fertility impairment and sterility (Ringborg et al., 2003; Jensen et al., 2011; Kesari et al., 2018). The ever-increasing trend of radiotherapy as a preferential treatment procedure for cancer patients with parallel rise in fertility problems requires greater attention towards research in this field. Our scientometric analysis revealed that a total of 14 articles were published in the year 2000, which has been increased to 50 articles in the year 2020. This 357% increase in per-year publications over the past two decades clearly signifies the growing interest and attention of the scientific research community to study the impact of radiotherapy on human fertility. Most importantly, 57.1% of all publications during the last 20 years were original articles, indicating a positive research growth towards this field. A majority (71.1%) of publications belongs to the field of medicine, which shows that the research may be focused on the clinical aspect of developing new drugs and medical procedures to minimize radiotherapy impact on fertility (Falls et al., 2018; Ahmad et al., 2019). Previous studies also support our hypothesis as research is progressing towards a way to minimize the effects of radiotherapy on fertility (Das et al., 2011; Barazzoul et al., 2020). Techniques such as intensity-modulated radiotherapy (IMRT) and proton radiotherapy may reduce the radiotherapy-related side effects (Wo and Viswanathan, 2009). However, more research and investigations are required to evaluate the effect of such radiotherapy techniques on male reproductive organs. These progressive clinical developments may benefit the young cancer survivors, as fertility problems are of great concern in the scientific community (Edge et al., 2006). In our analysis, the United States has been a leading country to conduct radiotherapy-related infertility research over the past two decades, which is in agreement with a previous report (Roldan-Valadez et al., 2019). In connection to this, Memorial Sloan-Kettering Cancer Center in New York and MD Anderson Cancer Center of The University of Texas were found to be the top two institutions conducting research in this field.

Global cancer incidence in men accounts for about 52% of the total cancer cases (World Cancer Resource Fund, 2021). Radiotherapy has harmful effects on the fertility potential of male cancer patients of reproductive age (Vakalopoulos et al., 2015). This has concerned andrologists and cancer researchers over the past few decades (Lee et al., 2006; Jeruss and Woodruff, 2009), which is well evident from the present scientometric study. Our results indicate a substantial growth of publication over the past two decades. Notably, the publication trend showed its peak in 2012 (n = 25; 500% increase) and 2019 (n = 22; 440% increase). Our scientometric analysis revealed that considerable amounts of publications (n = 70) were in the area of radiotherapy and its impact on the clinical conditions associated with male reproductive organs. Radiotherapy has been found to cause infertility in men by damaging the reproductive cells and by disrupting the balance of reproductive hormones (Mohan et al., 2019). Hence, it is important to carry out research to understand reproductive potential and functionality of men undergoing radiotherapy. This was in concordance with increase in the publication trend of radiotherapy and male reproductive research with collaboration observed between American, European and Asian countries, as reported in our study. In case of per-year publications during the last two decades, there was a 600% increase in the year 2019 (n = 18), with a drop of 33% in per year publication in the year 2020. The reason behind the decline of publications in 2020 (n = 12) may be due to the outstanding growth of research on the emergent coronavirus disease 2019 (COVID-19) pandemic. A bibliometric study revealed more than 1,500 publications on COVID-19 from 1 January 2020 to 8 March 2020 (Gong et al., 2020).

Radiotherapy has detrimental effects on semen and sperm quality (Singh et al., 2012; Vakalopoulos et al., 2015). Direct exposure of testis to radiation impairs spermatogenesis and exhibits germ cell loss (National Cancer Institute, 2020). Radiotherapy-induced sperm damage has often been linked with the production of reactive oxygen species (ROS), which are detrimental to sperm motility, count, and vitality (Agarwal et al., 2014; Alvarez et al., 1987; Awanti et al., 2010; Shi et al., 2012). Furthermore, ROS-induced mitochondrial dysfunction and lipid peroxidation may decrease sperm motility and count (Agarwal et al., 2014; Shi et al., 2012). These alarming facts have led to an overall increase in research interest on the impact of radiotherapy on semen parameters and male reproductive hormones. In the current study, per-year publication trend on radiotherapy impact on semen parameters has shown an increase of 367% in the year 2020 compared to 2000. About 60.6% of the total articles during the last 20 years were original articles, which signifies increasing research focus in this area. Despite a steady growth of research in this field, limited number of studies were published to understand the effect of radiotherapy on sperm abnormalities such as oligozoospermia (n = 5), reduction in motility (n = 8), viability (n = 8), semen volume (n = 6), change in normal morphology (n = 9), impairment of spermiogenesis (n = 2), and sperm DNA damage (n = 15). A pilot search (Supplementary Table S4) revealed that only a few studies, pertaining to the effect of radiotherapy on semen parameters/quality, were published from the time period 1996–2021, such as oligozoospermia (n = 9), reduction in sperm motility (n = 12), viability (n = 8), semen volume (n = 9), change in morphology (n = 9), and impaired spermiogenesis (n = 2). Radiotherapy also alters the levels of male reproductive hormones such as LH, FSH, and testosterone (Dueland et al., 2003). Our results show a very limited number of publications (n = 29) since 2000, which indicates that research related to the impact of radiotherapy on male reproductive hormones has not received a considerable focus in the last two decades. However, the publication trend in the last 10 years highlights that investigation of male reproductive hormones in patients undergoing radiotherapy is slowly gaining a gradual increase.

Nowadays, fertility preservation is being recommended to the patients undergoing radiotherapy (Martynov et al., 2020). Development and standardization of such protocols involves extensive research (Goossens et al., 2020). Despite the increase and decrease in publication throughout the last 20 years, our analysis shows an overall increase in the research related to radiotherapy and fertility preservation in men. Cryopreservation is one of the most common fertility preserving techniques, along with other options such as *in-vitro* maturation (IVM) of spermatogonia into spermatocytes or germ-cell transplantation into naïve testicular cells (Ajala et al., 2010). Testicular tissue cryopreservation and spermatogonial stem cell cryopreservation are emerging as novel fertility preservation techniques; however, these are still at experimental phases of study (Skaznik-Wikiel et al., 2015). Vitrification may be considered as an alternative approach to cryopreservation, which enables hydrated living cells to be cooled to cryogenic temperatures in the absence of ice (Fahy and Wowk, 2015). Despite the steady growth of fertility preservation related to radiotherapy, there is a very limited number of articles (n = 5) involving vitrification of germ cells of patients undergoing radiotherapy in the past two decades. This warrants extensive investigation to further understand the role of vitrification and to explore newer avenues in fertility preservation procedures. Current study results revealed that 52.7% of the total publications were original articles, which shows the studies are focused to identify the best way of preserving fertility among patients undergoing radiotherapy. It is important to highlight that fertility preservation, such as gonadoprotective strategies, and testicular tissue sampling and cryostorage, are applicable in prepubertal boys. However, there are many challenges that needs to be addressed before its clinical application (Wyns et al., 2021).

The high-energy ionizing radiation (IR) originating from the medical instruments during radiotherapy induces molecular changes in a dose-dependent manner (Roldan-Valadez et al., 2019). IR exposure increases the risk of genetic damage during spermatogenesis along with changes in DNA methylation pattern of sperm (Yauk et al., 2008). Since spermatogonia have a delayed DNA repair process compared to somatic cells, with each increased dose of radiation, the possibility of single-stranded DNA damage also increases, which may get transmitted to future generation (Wdowiak et al., 2019). However, relevant data on human spermatozoa is very limited, and in most of the cases, the animal data were extrapolated to humans (Meistrich, 2020). This was evident in our analysis, which showed that only two articles have been published in the area of radiotherapy investigating the molecular changes associated to male reproductive organs. Hence, there exists a potential research gap, and extensive research is warranted in this area of radiotherapy. Studying the impact of radiotherapy on the genomics and proteomics of spermatozoa and male reproductive organs may help in the identification of new prognostic, diagnostic, and therapeutic biomarkers which may aid in better management option(s).

## Conclusion

To our knowledge, this is the first scientometric study on radiotherapy and its impact on male fertility. Our scientometric analysis revealed a progressive research growth in the field of radiotherapy and male infertility in the last two decades. Research is predominantly focused on the effect of radiotherapy on semen parameters and fertility preservation. However, there is a need of extensive research evaluating the impact of radiotherapy on male reproductive hormones and the molecular changes associated with male reproductive organs. Furthermore, future research is warranted for the development and improvement of fertility-sparing therapeutic strategies for cancer patients.

## Data Availability

The original contributions presented in the study are included in the article/Supplementary Material
