# Supplementary material for: Recent Publication Trends in Radiotherapy and Male Infertility over Two Decades: A Scientometric Analysis
Source: Front Cell Dev Biol. 2022 May 12;10:877079. doi: 10.3389/fcell.2022.877079 (PMC9133602; doi:10.3389/fcell.2022.877079)
Supplement: Supplementary file 1 [file DataSheet1.pdf]

Supplementary Material

Supplementary Tables

Supplementary Table 1. Steps of the search procedure and keywords used to retrieve documents from SCOPUS database.

Steps	Content	Keyword string
**Step 1**	Publication trend on radiotherapy in humans	(A) AND TITLE-ABS-KEY ("human")
**Step 2**	Publication trend in radiotherapy in men and clinical conditions associated with male reproductive organs	(A) AND TITLE-ABS-KEY (“male” OR “man” OR “men”) AND TITLE-ABS-KEY(“prostate cancer” OR “prostate damage” OR “testicular cancer” OR “testicular tumour” OR “germ cell tumour” OR “germ cell cancer” OR “seminoma” OR “non seminoma” OR “testes* damage” OR “testis* damage” OR “ testis* injury” OR “testes* injury*” OR “epididymal cancer” OR “epididymis damage” OR “epididymis injury” OR “spermatocele” OR “vas deferens damage” OR “vas deferens injury” OR “penile cancer” OR “penis* damage” OR “penile injury” OR “penis injury” OR “scrotum damage” OR “scrotum injury*”OR “erectile dysfunction” OR “ED”)
**Step 3**	Publication trend in radiotherapy and male reproductive research	(A) AND TITLE-ABS-KEY ("seminal plasma" OR “seminal fluid” OR “semen” OR "sperm" OR "spermatozoa" OR "male fertility" OR "male infertility" OR "male sterility" OR "male subfertility")
**Step 4**	Publication trend in radiotherapy associated with semen parameters and male reproductive hormones	
	Semen parameters	(A) AND TITLE-ABS (“sperm*” OR “semen” OR “ejaculate” OR “seminal” OR “viscosity” OR “pH" OR “volume” OR “motil*” OR “viab*” OR “vital*” OR “morphology” OR “count” OR “low sperm count*” OR “azoospermia” OR “oligozoospermia” OR “teratozoospermia” OR “asthenozoospermia” OR “oligoasthenoteratozoospermia”)

	Male reproductive hormones	(A) AND TITLE-ABS-KEY (“male” OR “man” OR “men”) AND TITLE-ABS (“testosterone” OR “low testosterone* level” OR “luteinizing hormone” OR “follicle stimulating hormone” OR “LH” OR “FSH”)
**Step 5**	Radiotherapy and molecular changes in male reproductive organ	
	Genomic, proteomic, metabolomic, transcriptomic	(A) AND TITLE-ABS-KEY ("seminal plasma" OR “seminal fluid” OR "sperm" OR "spermatozoa" OR "male fertility" OR "male infertility" OR "male sterility" OR "male subfertility") AND TITLE-ABS (“genomic*” OR “transcriptomic*” OR “proteomic*” OR “metabolomic*” OR “microRNA*” OR “miRNA*” OR “epigenetic*”)
**Step 6**	Radiotherapy and fertility preservation in men	(A) AND TITLE-ABS-KEY (“male” OR “men” OR “man” OR “sperm” OR “spermatozoa” OR “testi* tissue” OR “micro-testi*”) AND TITLE-ABS-KEY (“cryopreservation” OR “cryoconservation” OR “testicular tissue harvesting” OR “sperm bank*” OR “vitrification” OR “vitri*”)

A= TITLE-ABS (“fertility”) AND TITLE-ABS (“radiotherap*” OR “radiation therap*” OR “radiation treat*”)

Supplementary Table 2. Criteria for exclusion of articles from further analysis

Steps	Content	Reasons for exclusion
Step 1	Radiotherapy in human	Consideration of only psychological aspects
		Chemotherapeutic studies
		Unrelated to radiotherapy
		Studies conducted in animal
		Articles not in English language
		Articles not specific to the study
		Studies conducted in children
		Studies not associated with any clinical condition/scenario
Step 2	Radiotherapy in men	Consideration of only psychological aspects
		Chemotherapeutic studies
		Unrelated to radiotherapy
		Studies conducted in animal
		Articles not in English
		Articles not specific to the study
		Studies conducted in children
		Studies conducted in women
		Studies not associated with any clinical condition/scenario
Step 3	Radiotherapy and male reproductive research	Not related to male reproductive research
		Studies conducted in animal
		Studies conducted in female
		Related to fertility preservation
		Unrelated to radiotherapy
		Committee opinion
Step 4	Radiotherapy on semen parameters and male reproductive hormones	Unrelated to sperm parameters
		Unrelated to reproductive hormone
		Studies conducted in animal
		Studies conducted in female
		Unrelated to radiotherapy
		Fertility preservation studies
		Chemotherapeutic studies
Step 5	Radiotherapy and fertility preservation in men	Unrelated to fertility preservation in men
		Studies conducted in animal
		Studies conducted in female
		Committee or ethical opinion
		Unrelated to radiotherapy
Step 6	Radiotherapy and molecular changes in male reproductive organ	Studies conducted in female

Supplementary Table 3. List of publications addressing the specific impact of radiotherapy on the aspects of semen parameters, reproductive hormones and fertility preservation during 2000-2021

Aspect	No. of studies	Specific Impact of Radiotherapy	No. of studies on the specific impact	References
**Semen parameters**	155	Sperm DNA damage	15	1-15
		Azoospermia	25	4, 16-39
		Oligozoospermia	5	4,23,24,34,38
		Reduction in sperm count	26	3,9,19,22,23,26,39-54,58-61
		Reduction in sperm motility	8	3,4,21,41,42,60-62
		Reduction in sperm viability	8	3,46,60,61,63-66
		Reduction in semen volume	6	16,51,66,67,68,69
		Change in sperm morphology	9	21,41,47,61,70-74
		Impairment of spermatogenesis	52	3,6,16,17,21,22,26-29,40,41,46,54,75-111,112
		Sperm Damage	41	113-153
		Impaired spermiogenesis	2	154,155
**Reproductive Hormones**	29	Reduction in testosterone level	20	54,63,67,81,73,102, 107,112,127,133,154,156 ,157,159,160-165
		Elevation of LH level	3	86,133,165
		Elevation of FSH level	10	26,73,67,69,85,86, 94,104,107,166,167
		Reduction in GnRH level	2	133, 164
**Fertility preservation**	169	Vitrification	5	108,168-171
		Cryopreservation of testicular tissue	100	8,10,12,13,16-20,23,25,27-30,40-43,46,49,51,53,54,58,59, 61,62,64,66,70,73,75,78-

			80,82-88,90,92,93,103,107-111,124,127,131,133-137,139,141-151,153,168-194
	Cryopreservation of sperm	89	3-7, 12,16,20,24,25,32,40,45,47,49,51,56,60,65,71,72,78,82,83,91,99,100,113,115,116,117,118,119,122,123,125,130,132,136,138,140,145,146,153,174,180,186,187,188,189,195-233

References for Supplementary Table 3

1. Frias S., Van Hummelen P., Meistrich M.L., Wyrobek A.J. Meiotic susceptibility for induction of sperm with chromosomal aberrations in patients receiving combination chemotherapy for Hodgkin lymphoma. Plos One (2021):15.

2. Haddad P., Karimimoghaddam Z., Farhan F., Esfahani M., Afkhami M., Amouzegar-Hashemi F. Delivered dose to scrotum in rectal cancer radiotherapy by thermoluminescence dosimetry comparing to dose calculated by planning software. Tehran Univ J Med (2014); 71(11): 707-712.

3. Paoli D., Pelloni M., Lenzi A., Lombardo F. Cryopreservation of sperm: Effects on chromatin and strategies to prevent them. Adv Exp Med Biol (2019); 1166: 149-167.

4. Desai N., Rambhia P. Cryopreservation of sperm for IVF: Semen samples and individual sperm. Principles of IVF Laboratory Practice: Optimizing Performance and Outcomes (2017); 112-118

5. Tamburrino L., Cambi M., Marchiani S., Manigrasso I., Degl'Innocenti S., Forti G., Maggi M., Baldi E., Muratori M. Sperm DNA fragmentation in cryopreserved samples from subjects with different cancers. Reprod Fertil Dev (2017) ;29(4):637-645.

6. Góngora-Rodríguez A., Capilla-González G., Parra-Forero L.Y. Fertility preservation in men with oncologic diseases. Reprod Med Biol. (2014); 13(4): 177–184.

7. Paoli D., Lombardo F., Lenzi A., Gandini L. Sperm cryopreservation: Effects on chromatin structure. Adv Exp Med Biol (2014); 791:137-50.

8. Budgell G.J., Cowan R.A., Hounsell A.R. Prediction of scattered dose to the testes in abdominopelvic radiotherapy. Clin Oncol (R Coll Radiol). 2001;13(2):120-5. doi: 10.1053/clon.2001.9234.

9. Romerius P., Ståhl O., Moëll C., Relander T., Cavallin-Ståhl E., Gustafsson H., Thapper K.L., Jepson K., Spanò M., Wiebe T., Giwercman Y.L., Giwercman A. Sperm DNA integrity in men treated for childhood cancer. Clin Cancer Res (2010) ;16(15):3843-50

10. Agarwal A., Ong C., Durairajanayagam D. Contemporary and future insights into fertility preservation in male cancer patients. Transl Androl Urol. 2014 Mar;3(1):27-40. doi: 10.3978/j.issn.2223-4683.2014.02.06.

11. Pasqualotto F.F., Agarwal A. Radiation efects on spermatogenesis. In Fertility Preservation in Male Cancer Patients (2010): 104-109

12. Cankut S., Dinc T., Cincik M., Ozturk G., Selam B. Evaluation of Sperm DNA Fragmentation via Halosperm Technique and TUNEL Assay Before and After Cryopreservation. Reproductive Sciences (2019); 26(12): 1575-1587.

13. Meseguer M., Santiso R., Garrido N., Fernandez J.L. The effect of cancer on sperm DNA fragmentation as measured by the sperm chromatin dispersion test. Fertil. Steril. (2008); 90(1): 225-227

14. O Donovan. An evaluation of chromatin condensation and DNA integrity in the spermatozoa of men with cancer before and after therapy. Andrologia (2005); 37(2):83-90.

15. Morris, ID. Sperm DNA damage and cancer treatment. Int J Androl (2002); 25(5): 255-261.

16. Pallotti F., Pelloni M., Faja F., Di Chiano S., Di Rocco A., Lenzi A., Lombardo F., Paoli D. Semen quality in non-Hodgkin lymphoma survivors: A monocentric retrospective study. Hum Reprod (2021);36(1):16-25

17. Gül M., Dong L., Wang D., Diri M.A., Andersen C.Y. Surrogate testes: Allogeneic spermatogonial stem cell transplantation within an encapsulation device may restore male fertility. Med Hypotheses (2020); 139:109634

18. Revel A., Revel-Vilk S. Pediatric fertility preservation: Is it time to offer testicular tissue cryopreservation? Mol Cell Endocrinol. (2008); 282(1-2):143-9.

19. Dittrich R., Lötz L., Hoffmann I., Beckmann M.W. Techniques of fertility protection: An update. Padiatrische Praxis (2017); 89(1): 121-130.

20. Nison L., Marcelli F., Rigot J.-M. Biopsy for testicular sperm extraction to preserve fertility in neoplastic context (onco-TESE): For whom and how? Progres en Urologie – FMC (2016); 26(3); F50-F55.

21. Paoli D., Rizzo F., Fiore G., Pallotti F., Pulsoni A., Annechini G., Lombardo F., Lenzi A., Gandini L. Spermatogenesis in Hodgkin's lymphoma patients: A retrospective study of semen quality before and after different chemotherapy regimens. Hum Repro (2016) 31(2):263-72

22. Ahmad G., Agarwal A. Ionizing radiation and male fertility. In Male Infertility: A Clinical Approach. 2016; 185-196.

23. Tran S., Boissier R., Perrin J., Karsenty G., Lechevallier E. Review of the Different Treatments and Management for Prostate Cancer and Fertility. Urology (2015) 86(5):936-41

24. Molnár Z., Benyó M., Bazsáné Kassai Z., Lévai I., Varga A., Jakab A. Influence of malignant tumors occurring in the reproductive age on spermiogenesis: Studies on patients with testicular tumor and lymphoma. Orv Hetil (2014) ;155(33):1306-11.

25. Gilbert K., Nangia A.K., Dupree J.M., Smith J.F., Mehta A. Fertility preservation for men with testicular cancer: Is sperm cryopreservation cost effective in the era of assisted reproductive technology? Urol Oncol: Semin Orig (2018); 36(3): 2-10

26. Brydoy M., Fosså S.D., Klepp O., Bremnes R.M., Wist E.A., Bjoro T., Wentzel-Larsen T., Dahl O. Sperm counts and endocrinological markers of spermatogenesis in long-term survivors of testicular cancer. Br J Cancer. (2012); 107(11): 1833–1839

27. Dohle G.R. Male infertility in cancer patients: Review of the literature. Int J Urol. (2010); 17(4):327-31

28. Gassei K., Schlatt St. Conservation of male fertility - Clinical and experimental methods. J. fur Reproduktionsmedizin und Endokrinologie (2009); 6(3):93-98

29. Schmidt K.L.T., Carlsen E., Andersen A.N. Fertility treatment in male cancer survivors. Int J Androl (2007); 30(4); 413-419.

30. Ragni G., Somigliana E., Restelli L., Salvi R., Arnoldi M., Paffoni A. Sperm banking and rate of assisted reproduction treatment: Insights from a 15-year cryopreservation program for male cancer patients. Cancer (2003); 97(7):1624-9

31. Pushcheck E. Male fertility preservation and cancer treatment. Cancer Treat Rev. (2004); 30(2):173-80. doi: 10.1016/j.ctrv.2003.07.005.

32. Tryde Schmidt K.L., Larsen E., Bangsbøll S., Meinertz H., Carlsen E., Andersen A.N. Assisted reproduction in male cancer survivors: fertility treatment and outcome in 67 couples. Hum Reprod (2004); 19-12:2806-2810

33. Sieniawski M, Reineke T, Josting A, Nogova L, Behringer K, Halbsguth T, et al. Assessment of male fertility in patients with Hodgkin's lymphoma treated in the German Hodgkin Study Group (GHSG) clinical trials. Ann Oncol. 2008 Oct;19(10):1795-801. doi: 10.1093/annonc/mdn376.

34. Dickstein R.J., Shetty G., Meistrich M.L. Application of spermatogenesis suppression therapies for fertility preservation. In Fertility preservation in male cancer patients (2010);203-212

35. Thomson A.B., Wallace W.H.B., Sklar C. Testicular function. In Late Effects of Childhood Cancer (2004): 239-256.

36. Barri P.N., Veiga A., Boada M., Solé M. Embryo cryopreservation as a fertility preservation strategy. In Principles and Practice of Fertility Preservation (2011): 279-282.

37. Rodríguez J.M.T., Ávila L.B., Zúñiga I.C., Rodríguez F.G., Hernández V.M.V. Fertility and testicular function in a patient with germ cell extragonadal tumor. A report of a case. Ginecol Obstet Mex (2004) 75(1):46-49

38. Gunn HM, Rinne I, Emilsson H, Gabriel M, Maguire AM, Steinbeck KS. Primary Gonadal Insufficiency in Male and Female Childhood Cancer Survivors in a Long-Term Follow-Up Clinic. J Adolesc Young Adult Oncol. (2016) 5(4):344-350. doi: 10.1089/jayao.2016.0007

39. Thomas C, Cans C, Pelletier R, De Robertis C, Hazzouri M, Sele B, et al. No long-term increase in sperm aneuploidy rates after anticancer therapy: sperm fluorescence in situ hybridization analysis in 26 patients treated for testicular cancer or lymphoma. Clin Cancer Res. (2004) 10(19):6535-43. doi: 10.1158/1078-0432.CCR-04-0582.

40. Allen C., Keane D., Harrison R.F. A survey of Irish consultants regarding awareness of sperm freezing and assisted reproduction. Irish Medical Journal (2003) 96(1):23-25

41. Ghasemi B., Mehrjardi A.M., Jones C., Ghasemi N. Semen analysis of subfertility caused by testicular carcinoma. Int J Reprod Biomed. (2020) 18(7): 539–550.

42. Walter J.R., Lohman M.E., Kundu S.D., Xu S. A new fertility risk rating system for surgical, radiotherapy, and chemotherapy interventions used in testicular cancer. Translational Cancer Research (2016); 5: S778-S781.

43. Tinkanen H. Maintaining fertility in connection with cancer therapy. Duodecim; lääketieteellinen aikakauskirja (2011); 127(5): 480-485.

44. Yildirim B.A., Onal C. Radiotherapy in the management of testicular cancers. In Principles and Practice of Urooncology: Radiotherapy, Surgery and Systemic Therapy. (2017) 123-140.

45. Xi, Q., Zhu, L., Hu, J. et al. Successful pregnancy in a seminoma patient after fertility preservation. Chin. -Ger. J. Clin. Oncol. (2012) 11, 615–617. Doi: 10.1007/s10330-012-1006-9

46. Jahnukainen K., Ehmcke J., Hou M., Schlatt S. Testicular function and fertility preservation in male cancer patients. Best Pract Res Clin Endocrinol Metab. (2011) 25(2):287-302. doi: 10.1016/j.beem.2010.09.007.

47. Wang J., Muller C., Lin K. Optimizing fertility preservation for pre- and postpubertal males with cancer. Semin Reprod Med. (2013) 31(4):274-85. doi: 10.1055/s-0033-1345275.

48. Baird D.C., Meyers G.J., Hu J.S. Testicular Cancer: Diagnosis and Treatment. Am Fam Physician. (2018) 97(4):261-268.

49. Wood G.J.A., Hayden R.P., Tanrikut C. Successful sperm extraction and live birth after radiation, androgen deprivation and surgical castration for treatment of metastatic prostate cancer. Andrologia (2017) 49(1): e12578. Doi: 10.1111/and.12578

50. Marquis A., Kuehni C.E., Strippoli M.-P.F., Kühne T., Brazzola P. Sperm analysis of patients after successful treatment of childhood acute lymphoblastic leukemia with chemotherapy. Pediatr Blood Cancer (2010) 55(1):208-10

51. Nalesnik J.G., Sabanegh Jr. E.S., Eng T.Y., Buchholz T.A. Fertility in men after treatment for stage 1 and 2A seminoma. Am J Clin Oncol. (2004) 27(6):584-8. doi: 10.1097/01.coc.0000135736.18493.dd.

52. Buchler T., Freeman A., Harland S. Contralateral intratubular germ cell neoplasia in a patient with testicular cancer. Nat Clin Pract Urol (2008) 5(5):284-8.

53. Shin T., Miyata A., Arai G., Okada H. Fertility in testicular cancer patients. Jpn. J. Cancer Chemother (2015); 42(3): 267-271.

54. Mydlo J.H., Lebed B. Does brachytherapy of the prostate affect sperm quality and/or fertility in younger men? Scand J Urol Nephrol (2004) 38(3):221-4.

55. Dubey P., Wilson G., Mathur K.K., Hagemeister F.B., Fuller L.M., Ha C.S., et al. Recovery of sperm production following radiation therapy for Hodgkin's disease after induction chemotherapy with mitoxantrone, vincristine, vinblastine, and prednisone (NOVP). Int J Radiat Oncol Biol Phys. (2000) 46(3):609-17. doi: 10.1016/s0360-3016(99)00338-7.

56. Astafyeva L.I., Zhukov O.B., Kadashev B.A., Klochkova I.S., Kobyakov G.L., Poddubsky A.A., et al. Preservation of fertility in men with brain tumors. Russian Journal of Hum Reprod (2019) 25(1):74-82. Doi: 10.17116/repro20192501174

57. Huyghe E., Thonneau P.F., Plante P. Fertility after testicular cancer. Andrologie (2001); 11(4):221-225.

58. Putowski L., Kuczyński W. Strategies for fertility preservation after anti-cancer therapy. Ginekologia polska (2003) 74(8): 638-645.

59. Rives N., Verhaeghe F., Di Pizio P., Rives A. Fertility preservation. La Revue du praticien (2018); 68(2); 213-219.

60. Lass A., Akagbosu F., Brinsden P. Sperm banking and assisted reproduction treatment for couples following cancer treatment of the male partner. Hum Reprod Update (2008); 7(4): 370-377.

61. Brydøy M., Fosså S.D., Klepp O., Bremnes R.M., Wist E.A., Wentzel-Larsen T., Dahl O. Paternity following treatment for testicular cancer. J Natl Cancer Inst (2015); 97(21): 1580-1588.

62. Nallella K.P., Sharma R.K., Said T.M., Agarwal A. Inter-sample variability in post-thaw human spermatozoa. Cryobiology (2004) 49(2):195-9

63. Bojanic N., Bumbasirevic U., Vukovic I., Bojanic G., Milojevic B., Nale D., et al Testis sparing surgery in the treatment of bilateral testicular germ cell tumors and solitary testicle tumors: A single institution experience. J Surg Oncol (2015)111(2):226-30

64. Fujita K., Tsujimura A. Fertility preservation for boys with cancer. Reprod Med Bio (2010); 9(4): 179-184.

65. El-Ahwany A., Samir H., Alahwany H. Using two different thawing temperatures and their effect on the motility recovery of human cryopreserved sperms in cancer patients. Middle East Fertil Soc J (2018) 23(4): 331-334.

66. Keene D.J.B., Sajjad Y., Makin G., Cervellione R.M. Sperm banking in the United Kingdom is feasible in patients 13 years old or older with cancer. J Urol (2012) 188(2); 594-597.

67. Krawczuk-Rybak M., Solarz E., Wojtkowska M., Wysocka J., Matysiak M., Gadomski A., et al. Gonadal function in young men after the treatment for Hodgkin lymphoma. Pediatr Endocrinol Diabetes Metab (2009)15(2):85-92

68. Li R.L., Zhang X.M. Advances in researches on inhibin B and male reproduction. Natl J Androl (2005); 11(4): 299-302

69. Schmiegelow M., Lassen S., Poulsen H.S., Schmiegelow K., Hertz H., Andersson A.-M., et al. Gonadal status in male survivors following childhood brain tumors. J Clin Endocrinol Metab (2001);86(6): 2446-2452 (128)

70. Stahl P.J., Stember D.S., Hsiao W., Schlegel P.N. Indications and strategies for fertility preservation in men. Clin Obstet Gynecol (2010); 53(4): 815-827.

71. De Luyk N., Pozzato G., Ricci G., Tamaro P., Manno M., Tomei F., Trombetta C. Pre-treatment and post-treatment fertility in young male patients affected by Hodgkin and non-Hodgkin lymphoma. Arch Ital Urol Androl (2012); 84(3):141-145.

72. Meseguer M., Molina N., García-Velasco J.A., Remohí J., Pellicer A., Garrido N. Sperm cryopreservation in oncological patients: A 14-year follow-up study. Fertil. Steril. (2006); 85(3): 640-645.

73. Van Casteren N.J., Boellaard W.P.A., Romijn J.C., Dohle G.R. Gonadal dysfunction in male cancer patients before cytotoxic treatment. Int. J. Androl. (2010); 33(1): 73-79

74. Ma L.H., Ding Q., Wang X. Advances in xenogeneic transplantation of spermatogonial stem cell and its bewilderment in clinical application. Natl. J. Androl. (2006)12(3):258-262.

75. David S., Orwig K.E. Spermatogonial Stem Cell Culture in Oncofertility. Urol Clin North Am (2020) 47(2):227-244

76. Lieng H., Chung P., Lam T., Warde P., Craig T. Testicular seminoma: Scattered radiation dose to the contralateral testis in the modern era. Pract Radiat Oncol (2018) 8(2): e57-e62

77. Haddad P., Karimi-Moghaddam Z., Esfahani M., Afkhami M., Farhan F., Amouzegar-Hashemi F. Thermoluminescence dosimetry of the dose received by scrotum and testes in radiotherapy of rectal cancer, compared to the point doses calculated by 3D-planning software. Phys Med. (2018) 45:143-145.

78. Vakalopoulos I., Dimou P., Anagnostou I., Zeginiadou T. Impact of cancer and cancer treatment on male fertility. Hormones (Athens). (2015) 14(4):579-89.

79. Schlatt S., Kliesch S. Male fertility protection. More than just sperm conservation? Gynakologische Endokrinologie (2012); 10(2):91-97.

80. De Lambert G., Poirot C., Guérin F., Brugières L., Martelli H. Preservation of fertility in children with cancer [La préservation de la fertilité dans les cancers de l'enfant]. Bull Cancer. (2015); 102(5):436-42.

81. Schouten N., Van Dalen T., Smakman N., Elias S.G., Van De Water C., Spermon R.J., Mulder L.S., Burgmans I.P.J. Male infertility after endoscopic Totally Extraperitoneal (Tep) hernia repair (Main): Rationale and design of a prospective observational cohort study. BMC Surg (2012); 12:7

82. Chiba K., Fujisawa M. Fertility preservation in men with cancer. Reprod Med Biol. (2014); 13(4): 177–184

83. Barak S. Fertility preservation in male patients with cancer. Best Pract Res Clin Obstet Gynaecol. (2019) 55:59-66

84. Ping P., Gu B.-H., Li P., Huang Y.-R., Li Z. Fertility outcome of patients with testicular tumor: Before and after treatment. Asian J Androl. (2014);16 (1): 107-111.

85. Howell S.J., Shalet S.M. Fertility preservation and management of gonadal failure associated with lymphoma therapy. Current oncology reports (2002); 4(5): 443-452

86. Mitchell R.T., Saunders P.T.K., Sharpe R.M., Kelnar C.J.H., Wallace W.H.B. Male fertility and strategies for fertility preservation following childhood cancer treatment. Endocr Dev (2009); 15:101-134

87. Magelssen H., Brydøy M., Fosså S.D. The effects of cancer and cancer treatments on male reproductive function. Nat Clinl Prac Urol (2006);3: 312–322.

88. Hamano I., Hatakeyama S., Ohyama C. Fertility preservation of patients with testicular cancer. Reprod. Med. Biol. (2017); 16(3): 240-251.

89. Radford J. Restoration of fertility after treatment for cancer. Horm Res. (2003); 59 Suppl 1:21-3.

90. Molnár Z., Berta E., Benyó M., Póka R., Kassai Z., Flaskó T., et al. Fertility of testicular cancer patients after anticancer treatment - Experience of 11 years. Pharmazie (2014); 69(6):437-441.

91. Thomson A.B., Critchley H.O.D., Kelnar C.J.H., Wallace W.H.B. Late reproductive sequelae following treatment of childhood cancer and options for fertility preservation. Best Pract Res Clin Endocrinol Metab. (2002);16(2):311-34.

92. Hennebicq S., des Cesos FF. Frequency and results of the use of cryopreserved semen. Andrologie (2004);14(4):398-403.

93. Schröder A.K., Diedrich K., Ludwig M. Strategies for preventing chemotherapy- and radiotherapy-induced gonadal damage. Am J Cancer (2004); 3(2): 97-117

94. Albers P. Germ-cell tumors and fertility. Reproduktionsmedizin (2000); 16(1):55-61.

95. Ginsberg J.P. Gonadotoxicity of cancer therapies in pediatric and reproductive-age males. In Oncofertility Medical Practice: Clinical Issues and Implementation (2012); 15-23

96. Ray K., Choudhuri R. Effects of Radiation on the Reproductive System. Reprod Dev Toxicol (2011); 291-299. Doi: 10.1016/B978-0-12-382032-7.10022-0

97. Kiserud C.E., Magelssen H., Fedorcsak P., Fosså S.D. Gonadal function after cancer treatment in adult men. Tidsskrift for den Norske Laegeforening (2008); 128(4):461-465 (

98. Brito V.N., Berger K., Mendonca B.B. Male hypogonadism: Childhood diagnosis and future therapies. Pediatric Health (2010); 4(5): 539-555. Doi: 10.2217/phe.10.50

99. Horne G, Atkinson A, Brison DR, Radford J, Yin JAL, Edi-Osagie ECO, et al. Achieving pregnancy against the odds: successful implantation of frozen–thawed embryos generated by ICSI using spermatozoa banked prior to chemo/radiotherapy for Hodgkin's disease and acute leukaemia: Case Report. Hum Reprod (2001) 16(1): 107–109, Doi: 10.1093/humrep/16.1.107

100. Feneux D. Hematologic malignancies: Fertility in males and couples I. Fertility in males and couples [Hémopathies malignes: Sexualité, fertilité et grossesse I. Fertilité des couples et fertilité masculine]. Hematologie (2001); 7(2): 115-120.

101. Efthekar M, Mohammadin F, Yousefnejad F, Molaei B, Aflatoonian A. Comparison of conventional IVF versus ICSI in non-male factor, normoresponder patients. Int J Reprod Biomed. (2012) 10(2): 131-136.

102. Tabei T., Yoshida M., Oouchi H., Suwa Y. A case of metachronous bilateral testicular tumor treated by partial orchiectomy and local radiation (2013); 67-12:979-982.

103. Wyns, C. Cryopreservation and transplantation of testicular tissue. Principles and Practice of Fertility Preservation (2011): 209-224

104. Grinspon R.P., Arozarena M., Prada S., Bargman G., Sanzone M., Morales Bazurto M., et al. Safety of standardised treatments for haematologic malignancies as regards to testicular endocrine function in children and teenagers. Hum Reprod. (2019) 34(12):2480-2494. doi: 10.1093/humrep/dez216.

105. Celik-Ozenci C. Spermatogenesis and testicular function. In Fertility Preservation: Emerging Technologies and Clinical Applications (2012); 245-260. Doi: 10.1007/9781441917836_19

106. De Palma A, Vicari E, Palermo I, D'Agata R, Calogero AE. Effects of cancer and anti-neoplastic treatment on the human testicular function. J Endocrinol Invest. (2000) 23(10):690-6. doi: 10.1007/BF03343795.

107. Vermeulen M., Del Vento F., Kanbar M., Ruys S.P.D., Vertommen D., Poels J., et al. Generation of organized porcine testicular organoids in solubilized hydrogels from decellularized extracellular matrix. Int. J. Mol. Sci. (2019) 20(21), 5476

108. Vermeulen M., Poels J., de Michele F., des Rieux A., Wyns C. Restoring Fertility with Cryopreserved Prepubertal Testicular Tissue: Perspectives with Hydrogel Encapsulation, Nanotechnology, and Bioengineered Scaffolds. Ann Biomed Eng. (2017); 45(7):1770-1781.

109. Sönmezer M., Özkavukçu S. Fertility preservation in females with malignant disease-1: Causes, clinical needs and indications. Turk J Hematol (2009); 26(3): 106-113.

110. Riboldi M., Marqués Marí A.I., Simón C. Stem cells and fertility preservation in males. In Fertility Preservation: Emerging Technologies and Clinical Applications (2012) 345-352

111. Mohazzab A., Heidari M., Salehkhou S., Jeddi-Tehrani M., Akhondi M.M. Fertility preservation in men after cancer treatment; a review article. J Reprod Infert (2011) 11(2): 73-84.

112. De Felice F., Marchetti C., Marampon F., Cascialli G., Muzii L., Tombolini V. Radiation effects on male fertility. Andrology (2019) 7(1): 7-2

113. Eghbali H, Papaxanthos-Roche A. The impact of lymphoma and treatment on male fertility. Expert Rev Hematol. 2010 Dec;3(6):775-88. doi: 10.1586/ehm.10.70.

114. Hughes PD. Partial orchidectomy for malignancy with consideration of carcinoma in situ. ANZ J Surg. (2006)76(1-2):92-4. doi: 10.1111/j.1445-2197.2006.03660.x.

115. Ogle S.K., Hobbie W.L., Carlson C.A., Meadows A.T., Reilly M.M., Ginsberg J.P. Sperm banking for adolescents with cancer. J Pediatr Oncol Nurs. (2008) 25(2):97-101. doi: 10.1177/1043454207311922.

116. Herrmann T., Thiede G., Trott K.-R., Voigtmann L. Nachkommen präkonzeptionell bestrahlter Eltern. Abschlussbericht einer Longitudinalstudie 1976-1994 und Empfehlungen zur Patientenberatung [Offsprings of preconceptionally irradiated parents. Final report of a longitudinal study 1976-1994 and recommendations for patients' advisory]. Strahlenther Onkol. (2004)180(1):21-30. German. doi: 10.1007/s00066-004-1223-4.

117. Casbas J.M.G., Domínguez M.C. Demand and utilization of a sperm bank in oncological patients: Cryopreservation of pre-chemotherapy, pre-radiotherapy and pre-surgical semen. Arch Espan Urol (2004); 57(9): 1.017-1.02

118. González Casbas J.M., Calderay Domínguez M. Requests for utilization of a semen bank among oncological patients. Semen cryopreservation prior to chemotherapy, radiotherapy and surgery. Arch Españ Urol (2004); 57(9): 1017-1020

119. Rötgens J., Van Belle S. The preservation of male fertility before, during and after cancer treatment: Current state of affairs. Tijdschr Geneeskd (2015); 71-12:797-806

120. Marec-Berard P., Dubois C., Giscard D'Estaing S., Pacquement H., Brugières L., Laurence V, et al. An information booklet on the semen freezing intended for adolescents and young adults treated for cancer and the evaluation of its use in paediatric oncology centres in France: Preliminary results. Oncologie (2013) 15(5):255-259

121. Garolla A., Pizzol D., Bertoldo A., Ghezzi M., Carraro U., Ferlin A., Foresta C. Testicular cancer and HPV semen infection. Front Endocrinol (Lausanne). (2012) 3:172. doi: 10.3389/fendo.2012.00172.

122. Jing Y.-X., Zhang L.-L., Li H.-X., Yue F., Wang N., Xue S.-L., Wang Y.-Q., Zhang X.-H. Fertility preservation in cancer patients. Reprod Dev Med (2021); 5(1):44-54 Doi: 10.4103/2096-2924.309789

123. Borgmann-Staudt A., Sommerhäuser G., Balcerek M. Fertility preservation in children and adolescents with cancer [Fertilitätserhalt bei Tumoren im Kindes- und Jugendalter]. Onkologeco (2021);27(5): 441-446. Doi: 10.1007/s00761-021-00908-9

124. Cobo, A. Clinical outcome after oocyte cryopreservation for elective fertility preservation. In Preventing Age Related Fertility Loss (2017): 117-124.

125. Dittrich R., Binder H., Mueller A., Maltaris T., Hoffmann I., Oppelt P.G, et al. Gonadal toxicity. Options for fertility preservation for patients facing the loss of gonadal function. Gynakologe (2008) 41(8): 613-620. Doi: 10.1007/s00129-008-2174-0

126. Verhaeghe F., Rives N. Sperm conservation in 2016: Who and how? Progres en Urologie - FMC (2017) 27(1): F9-F13. Doi: 10.1016/j.fpurol.2016.09.002

127. Dieckmann KP, Claßen J, Souchon R, Loy V. Management of testicular intraepithelial neoplasia (TIN) - A review on the foundation of evidence based medicine (EBM) [Therapie der testikulären intraepithelialen neoplasie (TIN) - Eine übersicht auf grundlage der evidenzbasierten medizin (EBM)]. Wien Klin Wochenschr (2001);113(1-2):7-14.

128. Hempel D., Chrenowicz R., Filipowski T., Wojtukiewicz M.Z., Sierko E. Testicular dose contributed by X-ray volume image-(XVI)-guided intensity-modulated radiotherapy (IMRT) in prostate cancer patients. Nowotwory (2020); 70(2): 47-53 Doi: 10.5603/NJO.2020.0012

129. Brusamolino E., Lunghi F., Orlandi E., Astori C., Passamonti F., Barate C., et al. Treatment of early-stage Hodgkin's disease with four cycles of ABVD followed by adjuvant radiotherapy: Analysis of efficacy and long-term toxicity. Haematologica (2000); 85(10): 1032-1039

130. Klepfish A., Shvidel L., Shtalrid M., Haran M., Berrebi A. High rate of response and low rate of complications in hodgkin's disease treated with mopp-ABV hybrid chemotherapy regimen with limited radiotherapy for areas of bulky disease: 15 years experience in a single institution. Blood (2000); 96(11 Part II):240b-241b.

131. Mazur-Roszak M., Tomczak P., Litwiniuk M., Markowska J. Oncology and infertility: Selected issues. Part II. Preservation of the reproductive function. Wspolczesna Onkologia (2005); 9(2): 65-68.

132. Horne G., Atkinson A.D., Pease E.H.E., Logue J.P., Brison D.R., Lieberman B.A. Live birth with sperm cryopreserved for 21 years prior to cancer treatment: Case report. Hum Reprod. (2004); 19(6): 1448-1449.

133. Catanzaro M., Piva L., Torelli T., Biasoni D., Stagni S., Milani A., et al. Funcion sparing surgery in uro-oncology: germ cell tumors of the testis. Urologia (2012);79 Suppl 19:15-9

134. Yuksel M.B., Gumus B., Özbek E., Nese N. A unique case of bilateral synchronous testicular tumor with concomitant bilateral diffuse intratubular germ cell neoplasia: Testis sparing surgery and local radiotherapy. Cur. Urol. (2013); 6(3): 165-168.

135. Brougham M.F.H., Wallace W.H.B. Subfertility in children and young people treated for solid and haematological malignancies. Brit. J. Haem. (2005); 131(2): 143-155.

136. Okada K., Fujisawa M. Recovery of spermatogenesis following cancer treatment with cytotoxic chemotherapy and radiotherapy. World J. Mens Health (2019); 37(2): 166-174.

137. Rives N., Macé B. Cryopreservation of testicular tissue in boys: How can the boy's fertility be preserved? Andrologie (2004); 14(4): 404-411

138. Rabah D.M., Wahdan I.H., Merdawy A., Abourafe B., Arafa M.A. Oncologists' knowledge and practice towards sperm cryopreservation in Arabic communities. Journal of Cancer Survivorship (2010); 4(3): 279-283.

139. Rives N., Milazzo J.-P., Sibert L., Liard-Zmuda A., Travers A., Arkoun B., et al. Fertility preservation in males. Medecine Therapeutique Medecine de la Reproduction, Gynecologie et Endocrinologie (2012); 14(2); 86-93.

140. Li Y., Zhang J., Zhang H., Liu B., Wang G., Cao M., et al. Importance and safety of autologous sperm cryopreservation for fertility preservation in young male patients with cancer. Medicine (2020); 99(15): e19589

141. Thomas C., Rousseaux S., De Robertis C., Pelletier R., Sele B., Hennebicq S. Male fertility and chromosome aneuploidy in sperm cells after radiotherapy or chemotherapy in patients with lymphoma or testicular cancer. Andrologie (2003); 13(4): 403-411.

142. Steinsvik E.A., Fosså S.D., Lilleby W., Eilertsen K. Fertility issues in patients with prostate cancer. BJU Int. (2008); 102(7): 793-795.

143. Colpi G.M., Contalbi G.F., Nerva F., Sagone P., Piediferro G. Testicular function following chemo-radiotherapy. Eur J Obstet Gynecol Reprod Biol (2004); 113: S2-S6.

144. Brougham M.F.H., Kelnar C.J.H., Sharpe R.M., Wallace W.H.B. Male fertility following childhood cancer: Current concepts and future therapies. Asian J Androl (2003); 5(4): 325-337.

145. Ortega C., Tournaye H. Impact of radiotherapy and chemotherapy on the testis. In Fertility Preservation: Emerging Technologies and Clinical Applications (2012): 261-270.

146. Anderson K.H., Romao R.L.P. Testicular tumors in children and adolescents: Long-term endocrine and fertility issues. Trans. Androl. Urol. (2020); 9(5): 2393-2399.

147. Huleihel M., Lunenfeld E. Approaches and technologies in male fertility preservation. Int. J. Mol. Sc. (2020); 21(15): 1-19.

148. Patel B., Rossi B.V. Preserving fertility in young patients with lymphoma: An overview. Blood and Lymphatic Cancer: Targets and Therapy (2015); 5:1-15

149. Isachenko E., Isachenko V., Sanchez R., Katkov I.I., Kreienberg R. Cryopreservation of spermatozoa: Old routine and new perspectives. Principles and Practice of Fertility Preservation (2011): 176-198.

150. Korte E., Balcerek M., Borgmann-Staudt A. Fertility impairment and possibilities of fertility protection following childhood cancer. Gynakologische Praxis (2017); 41(4):623-630.

151. Korte E., Balcerek M., Borgmann-Staudt A. Fertility impairment and possibilities of fertility protection following childhood cancer. Padiatrische Praxis (2016); 87(1): 61068

152. Ståhl O., Eberhard J., Jepson K., Spano M., Cwikiel M., Cavallin-Ståhl E., et al. The impact of testicular carcinoma and its treatment on sperm DNA integrity. Cancer. 2004 Mar 15;100(6):1137-44. doi: 10.1002/cncr.20068.

153. Poirot C., Sitbon L., Fortin A., Berthaut I., Jaudi S., Anastacio A., et al. Fertility and cancer. Presse Medicale (2013); 42(11): 1513-1520

154. Rendtorff R., Hohmann C., Reinmuth S., Müller A., Dittrich R., Beyer M., et al. Hormone and Sperm Analyses after Chemo- and Radiotherapy in Childhood and Adolescence. Klin Padiatr. (2010) 222(3):145-9. doi: 10.1055/s-0030-1249658. (101)

155. Aslam I., Fishel S., Moore H., Dowell K., Thornton S. Fertility preservation of boys undergoing anti-cancer therapy: A review of the existing situation and prospects for the future. Hum Reprod (2000);15(10):

156. Raison N., Warrington J., Alnajjar H.M., Muneer A., Ahmed K. The role of partial orchidectomy in the management of small testicular tumours: Fertility and endocrine function. Andrology (2020) 8(5):988-995.

157. Bruheim K, Svartberg J, Carlsen E, Dueland S, Haug E, Skovlund E, Tveit KM, Guren MG. Radiotherapy for rectal cancer is associated with reduced serum testosterone and

increased FSH and LH. Int J Radiat Oncol Biol Phys. (2008) 70(3):722-7. doi: 10.1016/j.ijrobp.2007.10.043.

158. Gunn HM, Rinne I, Emilsson H, Gabriel M, Maguire AM, Steinbeck KS. Primary Gonadal Insufficiency in Male and Female Childhood Cancer Survivors in a Long-Term Follow-Up Clinic. J Adolesc Young Adult Oncol. (2016) 5(4):344-350. doi: 10.1089/jayao.2016.0007.

159. Sargos P., Ferretti L., Henriques de Figueiredo B., Cornelis F., Belhomme S., Dallaudière B., et al. Radiotherapy after testicular-sparing surgery for bilateral or monorchide testicular tumours: An innovative approach. Cancer Radiother (2013);17(4):317-22

160. Grant and Ramasamy, The pituitary gland and erectile dysfunction: Causes, investigation and management. In Erectile Dysfunction: Causes, Risk Factors and Management (2012): 129-143

161. Huddart RA, Norman A, Moynihan C, Horwich A, Parker C, Nicholls E, Dearnaley DP. Fertility, gonadal and sexual function in survivors of testicular cancer. Br J Cancer. (2005) 93(2):200-7. doi: 10.1038/sj.bjc.6602677.

162. Trabado S., Maione L., Brailly-Tabard S., Young J. Male acquired hypogonadotropic hypogonadism: Diagnosis and treatment. Ann Endocrinol (Paris) (2012) 73(2):141-6.

163. Brito V.N., Berger K., Mendonca B. Male hypogonadism: Childhood diagnosis and future therapies. Pediatric Health (2010) 4(5): 539-555.

164. van der Kaaij MA, Heutte N, Le Stang N, Raemaekers JM, Simons AH, Carde P, et al. European Organisation for Research and Treatment of Cancer: EORTC Lymphoma Group; Groupe d'Etude des Lymphomes de l'Adulte. Gonadal function in males after chemotherapy for early-stage Hodgkin's lymphoma treated in four subsequent trials by the European Organisation for Research and Treatment of Cancer: EORTC Lymphoma Group and the Groupe d'Etude des Lymphomes de l'Adulte. J Clin Oncol. (2007) 25(19):2825-32. doi: 10.1200/JCO.2006.10.2020.

165. Schlatt S, Nieschlag E. Keimzelltransplantation als Methode zur Fertilitätserhaltung bei onkologischen Patienten [Germ cell transplantation as a tool for fertility preservation of oncological patients]. Klin Padiatr. (2001) 213(4):250-4. German. doi: 10.1055/s-2001-16856.

166. Pfitzer C., Chen C.M., Wessel T., Keil T., Sörgel A., Langer T., et al. Dynamics of fertility impairment in childhood brain tumour survivors. J Cancer Res Clin Oncol (2014); 140(10):1759-67

167. Ebert A.K., Bals-Pratsch M., Seifert B., Reutter H., Rösch W.H. Genital and reproductive function in males after functional reconstruction of the exstrophy-epispadias complex--long-term results. Urology. (2008) 72(3):566-9; discussion 569-70. doi: 10.1016/j.urology.2007.11.166.

168. Shah T.A., Keye Jr. W.R. Fertility: Tissue and cell banking overview. Clin Lab Med. (2005) 25(3):557-69.

169. Rousset-Jablonski C., Chevillon F., Dhedin N., Poirot C. Fertility preservation in adolescents and young adults with cancer. J Clin Oncol. (2010); 28(32):4831-41

170. Youssry M., Schöpper B., Schultze-Mosgau A., Von Otte S., Griesinger G., Diedrich K., et al. Ongoing twin pregnancy after transfer of vitrified oocyte injected with sperm recovered from cryopreserved testicular tissue. Middle East Fertil. Soc. J. (2007);12(3):213-215

171. Gholami M., Ahmadi S.A.Y., Abaszadeh A., Khaki A. Protective effects of melatonin and ghrelin on spermatogenesis: A narrative review of the literature. Int J Reprod Biomed (2017);15(5):265-272

172. Blackhall, F., Atkinson, A., Maaya, M, Ryder WDJ, Horne G, Brison DR, et al. Semen cryopreservation, utilisation and reproductive outcome in men treated for Hodgkin's disease. Br J Cancer (2002) 87, 381–384 .

173. Schmidt K.T., Andersen A.N., Loft A., Ernst E., Andersen C.Y. Cancer and infertility. Ugeskrift for Laeger (2012); 174(41): 2455: 2459.

174. Ji Y. Fertility preservation for adolescent survivors with malignant tumor. Tumor (2013); 33(10): 935-938.

175. Kaneva K., Erickson L., Rowell E., Badawy S.M. Fertility preservation education for pediatric hematology-oncology fellows, faculty and advanced practice providers: a pilot study. Pediatr Hematol Oncol. (2021); 24:1-6

176. Fallat M.E., Hutter J. Preservation of fertility in pediatric and adolescent patients with cancer. Pediatrics. (2008);121(5): e1461-9.

177. David S., Orwig K.E. Fertility preservation in cancer patients. Biol Mamm Sperm (2017): 315-341.

178. von Horn K., Depenbusch M., Schultze-Mosgau A., Neumann K., Griesinger G. Fertility preservation in oncology patients. Onkology (2017); 23(11): 943-950.

179. Levine, J. Fertility preservation in children and adolescents with cancer. Minerva Pediatrica (2011); 63(1):49-59.

180. Redig A.J., Brannigan R., Stryker S.J., Woodruff T.K., Jeruss J.S. Incorporating fertility preservation into the care of young oncology patients. Cancer (2011); 117(1):1-10

181. Di Pietro M.L., Teleman A.A. Cryopreservation of testicular tissue in pediatrics: Practical and ethical issues. J Matern-Fetal Neonatal Med (2013); 26(15): 1524-1527

182. Shin D., Lo K.C., Lipshultz L.I. Treatment options for the infertile male with cancer. J Natl Cancer Inst. Monographs (2005); 34:48-50.

183. Gul M., Hildorf S., Dong L., Thorup J., Hoffmann E.R., Jensen C.F.S., et al. Review of injection techniques for spermatogonial stem cell transplantation. Hum. Reprod. Update (2020); 26(3): 368-391.

184. Martin J.R., Patrizio P. Options for fertility preservation in pediatric populations undergoing cancer chemotherapy. Pediatric Endocrinol Rev (2009); 6(2); 306-314.

185. Vermeulen M., Giudice M.-G., Del Vento F., Wyns C. Role of stem cells in fertility preservation: Current insights. Stem Cells Cloning (2012); 12: 27-48.

186. Gurgan T., Salman C., Demirol A. Pregnancy and Assisted Reproduction Techniques in Men and Women after Cancer Treatment. Placenta (2008); 29(2): 152-159.

187. Beretta G. Iatrogenic infertility. In Clinical Management of Male Infertility (2015):145-152.

188. Bazeos A., Al-Shawaf T., Lower A., Wilson C., Gedis Grudzinskas J. Preservation of reproductive capacity of cancer patients. Reprod Technol (2000); 10(1): 42-49.

189. Oldenburg J., Fosså S.D. Long-term toxicity after therapy for testicular cancer with special focus on sexual disorders. Urologe - Ausgabe A (2004); 48(4): 372-376.

190. Wallace W.H.B. Oncofertility and preservation of reproductive capacity in children and young adults. Cancer (2011); 117(20): 2301-2310.

191. Castellotti D.S., Cambiaghi A.S. Fertility preservation for oncologic patients. Rev. Bras. Hematol. Hemoter. (2008); 30(5): 406-410.

192. de Lambert G., Poirot C., Guérin F., Brugières L., Martelli H. Preservation of future fertility in pediatric patients with cancer. J Visc Surg (2018); 155: S41-S46.

193. Ginsberg J.P. New advances in fertility preservation for pediatric cancer patients. Curr Opin Pediatr. (2011) 23(1):9-13. doi: 10.1097/MOP.0b013e3283420fb6.

194. Dittrich R., Lotz L., Hackl J., Nichols-Burns S., Hildebrandt T., Schneider H., Hoffmann I., Beckmann M.W.

195. Astaf'eva L.I., Zhukov O.B., Kadashev B.A., Klochkova I.S., Kobyakov G.L., Poddubskiy A.A., Kalinin P.L. Reproductive disorders and preservation of fertility in males with benign and malignant brain tumors. Zh. Vopr. Nejrokhir. Im. N.N. Burdenko (2019); 83(2): 59-65.

196. Kyono K. Fertility preservation. J Mamm. Ova Res. (2013); 30(3): 101-108.

197. Vallone R., Buonfantino C., Conforti A., De Rosa P., Cariati F., Picarelli S., et al. An update about oncofertility. Biochimica Clinica (2017); 41(4): 322-334.

198. Lockwood G. Oocyte cryopreservation: time to come in out of the cold... Women's Health Medicine (2006); 3(3): 128-129.

199. Puscheck E., Philip P.A., Jeyendran R.S. Male fertility preservation and cancer treatment. Cancer Treat Rev. 2004 Apr;30(2):173-80. doi: 10.1016/j.ctrv.2003.07.005.

200. Amirjannati N., Sadeghi M., Hosseini Jadda S.H., Ranjbar F., Kamali K., Akhondi M.A. Evaluation of semen quality in patients with malignancies referred for sperm banking before cancer treatment. Andrologia. (2011) 43(5):317-20. doi: 10.1111/j.1439-0272.2010.01077.x.

201. Pauli S.A., Berga S.L., Shang W., Session D.R. Current status of the approach to assisted reproduction. Pediatr Clin North Am. (2009) 56(3):467-88, Table of Contents. doi: 10.1016/j.pcl.2009.04.001.

202. Moss J.L., Choi A.W., Fitzgerald Keeter M.K., Brannigan R.E. Male adolescent fertility preservation. Fertil Steril. (2016) 105(2):267-73. doi: 10.1016/j.fertnstert.2015.12.002.

203. Grigg A. he impact of conventional and high-dose therapy for lymphoma on fertility. Clin Lymphoma. (2004)5(2):84-8. doi: 10.3816/clm.2004.n.013.

204. Guérin J.-F. Cryoconservation de tissu testiculaire chez le garçon prépubère: indications et faisabilité [Testicular tissue cryoconservation for prepubertal boy: indications and feasibility]. Gynecol Obstet Fertil. (2005) 33(10):804-8. French. doi: 10.1016/j.gyobfe.2005.07.033.

205. Preface. Fertility Cryopreservation (2010); xi-xii. Doi: 10.1017/CBO9780511730207.001

206. Sirohi B., Rohatgi T.B., Lambertini M. Oncofertility and COVID-19-cancer does not wait. Ecancermedicalscience. (2020)14:ed101. doi: 10.3332/ecancer.2020.ed101.

207. Kim S.-Y., Kim S.K., Lee J.R., Woodruff T.K. oward precision medicine for preserving fertility in cancer patients: existing and emerging fertility preservation options for women. J Gynecol Oncol. (2016) 27(2):e22. doi: 10.3802/jgo.2016.27.e22.

208. Anderson R.A. Fertility preservation techniques: laboratory and clinical progress and current issues. Reproduction. (2008)136(6):667-9. doi: 10.1530/REP-08-0270.

209. Gunasheela D., Gunasheela S. Strategies for fertility preservation in young patients with cancer: a comprehensive approach. Indian J Surg Oncol. (2014) 5(1):17-29. doi: 10.1007/s13193-014-0291-x.

210. Leyvraz Recrosio C., Vaucher L., Primi M.-P. Fertility preservation and cancer in the male [Préservation de la fertilité masculine et cancer]. Revue Medicale Suisse (2012); 8(365): 2335-2339

211. Vázquez M.R., García M.G., Piñón M.L., Rodríguez M.C., Cancelo C.B., Mallo R.O.F., et al. Fertility preservation program in cancer patientes [Programa de preservación de la fertilidad en pacientes oncológicos]. Revista Iberoamericana de Fertilidad y Reproduccion Humana (2015);32(4): 35-44

212. Küçük M., Bolaman A.Z., Yavaşoğlu I., Kadiköylü G. Fertility-preserving treatment options in patients with malignant hematological diseases. Turk J Haematol. (2012) 29(3):207-16. doi: 10.5505/tjh.2012.72681.

213. Zhao H., Jin L., Li Y., Zhang C., Wang R., Li Y., et al. Oncofertility: What can we do from bench to bedside? Cancer Lett. (2019) 442:148-160. doi: 10.1016/j.canlet.2018.10.023.

214. Dittrich R., Maltaris T., Hoffmann I., Oppelt P.G., Beckmann M.W., Mueller A. Fertility preservation in cancer patients. Minerva Ginecologica (2010); 62(1): 63-80.

215. Klipstein S., Fallat M.E., Savelli S., Katz A.L., MacAuley R.C., Mercurio M.R., COMMITTEE ON BIOETHICS; SECTION ON HEMATOLOGY/ONCOLOGY; SECTION ON SURGERY. Fertility Preservation for Pediatric and Adolescent Patients With Cancer: Medical and Ethical Considerations. Pediatrics. (2020) 145(3):e20193994. doi: 10.1542/peds.2019-3994.

216. Alexandroni, H., Shoham, G., Levy-Toledano, R. et al. Fertility preservation from the point of view of hematopoietic cell transplant specialists—a worldwide-web-based survey analysis. Bone Marrow Transplant (2019). 54, 1747–1755 doi:10.1038/s41409-019-0519z

217. Kort J.D., Eisenberg M.L., Millheiser L.S., Westphal L.M. Fertility issues in cancer survivorship. CA Cancer J Clin. (2014) 64(2):118-34. doi: 10.3322/caac.21205.

218. Ronn R, Holzer HEG. Oncofertility in Canada: The Impact of Cancer on Fertility. Current Oncology. (2013) 20(4):338-344. Doi:10.3747/co.20.1358

219. Botha M.H., Kruger T.F. A review of the incidence and survival of childhood and adolescent cancer and the effects of treatment on future fertility and endocrine development. S. Afr. J. Obstet. Gynaecol. (2012); 18(2): 48-53

220. Wilkes S., Coulson S., Crosland A., Rubin G., Stewart J. Experience of fertility preservation among younger people diagnosed with cancer. Hum Fertil (Camb). (2010) 13(3):151-8. doi: 10.3109/14647273.2010.503359.

221. Maltaris T., Koelbl H., Seufert R., Kiesewetter F., Beckmann M.W., Mueller A., Dittrich R. Gonadal damage and options for fertility preservation in female and male cancer survivors. Asian J. Androl. (2006); 8(5): 515-533.

222. Muñoz M., Santaballa A., Seguí M.A., Beato C., de la Cruz S., Espinosa J., et al. SEOM Clinical Guideline of fertility preservation and reproduction in cancer patients. Clin Transl Oncol. (2016) 18(12):1229-1236. doi: 10.1007/s12094-016-1587-9.

223. Donnez J., Kim S.S. Principles and practice of fertility preservation. Principles and Practice of Fertility Preservation (2011); 1-549. Doi: 10.1017/CBO9780511921896

224. Biedka M., Kuźba-Kryszak T., Nowikiewicz T., Zyromska A. Fertility impairment in radiotherapy. Contemp Oncol (Pozn). (2016) 20(3):199-204. doi: 10.5114/wo.2016.57814.

225. Amzai G., Stojanovic A. Preservation of fertility and of reproduction ability in lymphoma patients. Maced. J. Med. Sci. (2013);6(2)

226. Tamás S., Róbert P. Onkofertilitás és kezelési lehetőségei. Irodalmi áttekintés [Oncofertility and therapeutic modalities. Survey of literature]. Orv Hetil. (2017)158(18):683-691. Hungarian. doi: 10.1556/650.2017.30730.

227. Ginsberg JP. Educational paper: The effect of cancer therapy on fertility, the assessment of fertility and fertility preservation options for pediatric patients. Eur J Pediatr. (2011) 170(6):703-708.

228. Hardy K., Wright C., Rice S., Tachataki M., Roberts R., Morgan D., Spanos S., Taylor D. Future developments in assisted reproduction in humans. Reproduction. (2002) 123(2):171-83. doi: 10.1530/rep.0.1230171.

229. Overbeek A., van den Berg M., Louwé L., Wendel E., ter Kuile M., Kaspers G., Stiggelbout A., van Dulmen-Den Broeder E., Hilders C. Practice, attitude and knowledge of Dutch paediatric oncologists regarding female fertility. Neth J Med (2014); 72(5): 264-270.

230. Vassilakopoulou M., Boostandoost E., Papaxoinis G., de La Motte Rouge T., Khayat D., Psyrri A. Anticancer treatment and fertility: Effect of therapeutic modalities on reproductive system and functions. Crit Rev Oncol Hematol. (2016) 97:328-334. Doi: 10.1016/j.critrevonc.2015.08.002

231. Brusamolino E., Bacigalupo A., Barosi G., Biti G., Gobbi P.G., Levis A., et al. Classical Hodgkin's lymphoma in adults: guidelines of the Italian Society of Hematology, the Italian Society of Experimental Hematology, and the Italian Group for Bone Marrow Transplantation on initial work-up, management, and follow-up. Haematologica. (2009);94(4):550-65. doi: 10.3324/haematol.2008.002451.

232. Xu R., Centola G.M., Tanrikut C. Genitourinary cancer patients have worse baseline semen parameters than healthy sperm bankers. Andrology (2019)7(4):449-453

233. Onofre J., Baert Y., Faes K., Goossens E. Cryopreservation of testicular tissue or testicular cell suspensions: A pivotal step in fertility preservation. Hum Reprod Update. (2016) 22(6): 744–761

Supplementary Table 4. List of publications addressing the specific impact of radiotherapy on the aspects of semen parameters, reproductive hormones and fertility preservation during 1966-2021

Aspect	No. of studies	Specific Impact of Radiotherapy	No. of studies on the specific impact	References
**Semen parameters**	205	Sperm DNA damage	15	1-15
		Azoospermia	44	4, 16-58
		Oligozoospermia	9	4,23,24,34,38,47,48,59,60
		Reduction in sperm count	40	3,9,19,22,23,26,39,40,43,45,47,48,50,61-75,79-90
		Reduction in sperm motility	12	3,4,21,45,62,63,81,82,84,85,91,92
		Reduction in sperm viability	8	3,67,81,82,93-96
		Reduction in semen volume	9	16,72,96-102
		Change in sperm morphology	9	21,62,68,82,103-107
		Impairment of spermatogenesis	81	3,6,16,17,21,22,26-29,40,41,45,48,50-52,55,56,61,62,67,75,85-87,89,108-161
		Sperm Damage	42	162-203
		Impaired spermiogenesis	2	204-205
**Reproductive Hormones**	49	Reduction in testosterone level	35	43,45,47,50,56,59,75,86,87,89,93,100,114,106,135, 140,145,146,151-153,176,182,204,206,207,209,210-217
		Elevation of LH level	6	59, 119,149,182,215,216
		Elevation of FSH level	16	26,52,56,58,59,100,102,118,119, 127,137,140,149,218-220
		Reduction in GnRH level	2	182, 214

**Fertility preservation**	169	Vitrification	5	108,168-171
		Cryopreservation of testicular tissue	100	8,10,12,13,16-20,23,25,27-30,40-43,46,49,51,53,54,58,59,61,62,64,66,70,73,75,78-80,82-88,90,92,93,103,107-111,124,127,131,133-137,139,141-151,153,168-194
		Cryopreservation of sperm	89	3-7, 12,16,20,24,25,32,40,45,47,49,51,56,60,65,71,72,78,82,83,91,99,100,113,115,116,117,118,119,122,123,125,130,132,136,138,140,145,146,153,174,180,186,187,188,189,195-233

References for Supplementary Table 4

1. Frias S., Van Hummelen P., Meistrich M.L., Wyrobek A.J. Meiotic susceptibility for induction of sperm with chromosomal aberrations in patients receiving combination chemotherapy for Hodgkin lymphoma. Plos One (2021):15.

2. Haddad P., Karimimoghaddam Z., Farhan F., Esfahani M., Afkhami M., Amouzegar-Hashemi F. Delivered dose to scrotum in rectal cancer radiotherapy by thermoluminescence dosimetry comparing to dose calculated by planning software. Tehran Univ J Med (2014); 71(11): 707-712.

3. Paoli D., Pelloni M., Lenzi A., Lombardo F. Cryopreservation of sperm: Effects on chromatin and strategies to prevent them. Adv Exp Med Biol (2019); 1166: 149-167.

4. Desai N., Rambhia P. Cryopreservation of sperm for IVF: Semen samples and individual sperm. Principles of IVF Laboratory Practice: Optimizing Performance and Outcomes (2017); 112-118

5. Tamburrino L., Cambi M., Marchiani S., Manigrasso I., Degl'Innocenti S., Forti G., Maggi M., Baldi E., Muratori M. Sperm DNA fragmentation in cryopreserved samples from subjects with different cancers. Reprod Fertil Dev (2017) ;29(4):637-645.

6. Góngora-Rodríguez A., Capilla-González G., Parra-Forero L.Y. Fertility preservation in men with oncologic diseases. Reprod Med Biol. (2014); 13(4): 177–184.

7. Paoli D., Lombardo F., Lenzi A., Gandini L. Sperm cryopreservation: Effects on chromatin structure. Adv Exp Med Biol (2014); 791:137-50.

8. Budgell G.J., Cowan R.A., Hounsell A.R. Prediction of scattered dose to the testes in abdominopelvic radiotherapy. Clin Oncol (R Coll Radiol). 2001;13(2):120-5. doi: 10.1053/clon.2001.9234. (83)

9. Romerius P., Ståhl O., Moëll C., Relander T., Cavallin-Ståhl E., Gustafsson H., Thapper K.L., Jepson K., Spanò M., Wiebe T., Giwercman Y.L., Giwercman A. Sperm DNA integrity in men treated for childhood cancer. Clin Cancer Res (2010) ;16(15):3843-50

10. Agarwal A., Ong C., Durairajanayagam D. Contemporary and future insights into fertility preservation in male cancer patients. Transl Androl Urol. 2014 Mar;3(1):27-40. doi: 10.3978/j.issn.2223-4683.2014.02.06. (84 XL)

11. Pasqualotto F.F., Agarwal A. Radiation efects on spermatogenesis. In Fertility Preservation in Male Cancer Patients (2010): 104-109 (4)

12. Cankut S., Dinc T., Cincik M., Ozturk G., Selam B. Evaluation of Sperm DNA Fragmentation via Halosperm Technique and TUNEL Assay Before and After Cryopreservation. Reproductive Sciences (2019); 26(12): 1575-1587.

13. Meseguer M., Santiso R., Garrido N., Fernandez J.L. The effect of cancer on sperm DNA fragmentation as measured by the sperm chromatin dispersion test. Fertil. Steril. (2008); 90(1): 225-227

14. O Donovan. An evaluation of chromatin condensation and DNA integrity in the spermatozoa of men with cancer before and after therapy. Andrologia (2005); 37(2):83-90. (149)

15. Morris, ID. Sperm DNA damage and cancer treatment. Int J Androl (2002); 25(5): 255-261. (150)

16. Pallotti F., Pelloni M., Faja F., Di Chiano S., Di Rocco A., Lenzi A., Lombardo F., Paoli D. Semen quality in non-Hodgkin lymphoma survivors: A monocentric retrospective study. Hum Reprod (2021);36(1):16-25

17. Gül M., Dong L., Wang D., Diri M.A., Andersen C.Y. Surrogate testes: Allogeneic spermatogonial stem cell transplantation within an encapsulation device may restore male fertility. Med Hypotheses (2020); 139:109634

18. Revel A., Revel-Vilk S. Pediatric fertility preservation: Is it time to offer testicular tissue cryopreservation? Mol Cell Endocrinol. (2008); 282(1-2):143-9.

19. Dittrich R., Lötz L., Hoffmann I., Beckmann M.W. Techniques of fertility protection: An update. Padiatrische Praxis (2017); 89(1): 121-130.

20. Nison L., Marcelli F., Rigot J.-M. Biopsy for testicular sperm extraction to preserve fertility in neoplastic context (onco-TESE): For whom and how? Progres en Urologie – FMC (2016); 26(3); F50-F55.

21. Paoli D., Rizzo F., Fiore G., Pallotti F., Pulsoni A., Annechini G., Lombardo F., Lenzi A., Gandini L. Spermatogenesis in Hodgkin's lymphoma patients: A retrospective study of semen quality before and after different chemotherapy regimens. Hum Repro (2016) ;31(2):263-72

22. Ahmad G., Agarwal A. Ionizing radiation and male fertility. In Male Infertility: A Clinical Approach. 2016; 185-196.

23. Tran S., Boissier R., Perrin J., Karsenty G., Lechevallier E. Review of the Different Treatments and Management for Prostate Cancer and Fertility. Urology (2015);86(5):936-41

24. Molnár Z., Benyó M., Bazsáné Kassai Z., Lévai I., Varga A., Jakab A. Influence of malignant tumors occurring in the reproductive age on spermiogenesis: Studies on patients with testicular tumor and lymphoma. Orv Hetil (2014) ;155(33):1306-11.

25. Gilbert K., Nangia A.K., Dupree J.M., Smith J.F., Mehta A. Fertility preservation for men with testicular cancer: Is sperm cryopreservation cost effective in the era of assisted reproductive technology? Urol Oncol: Semin Orig (2018); 36(3): 2-10

26. Brydoy M., Fosså S.D., Klepp O., Bremnes R.M., Wist E.A., Bjoro T., Wentzel-Larsen T., Dahl O. Sperm counts and endocrinological markers of spermatogenesis in long-term survivors of testicular cancer. Br J Cancer. (2012); 107(11): 1833–1839

27. Dohle G.R. Male infertility in cancer patients: Review of the literature. Int J Urol. (2010); 17(4):327-31

28. Gassei K., Schlatt St. Conservation of male fertility - Clinical and experimental methods. J. fur Reproduktionsmedizin und Endokrinologie (2009); 6(3):93-98

29. Schmidt K.L.T., Carlsen E., Andersen A.N. Fertility treatment in male cancer survivors. Int J Androl (2007); 30(4); 413-419.

30. Ragni G., Somigliana E., Restelli L., Salvi R., Arnoldi M., Paffoni A. Sperm banking and rate of assisted reproduction treatment: Insights from a 15-year cryopreservation program for male cancer patients. Cancer (2003); 97(7):1624-9

31. Pushcheck E. Male fertility preservation and cancer treatment. Cancer Treat Rev. (2004); 30(2):173-80. doi: 10.1016/j.ctrv.2003.07.005.

32. Tryde Schmidt K.L., Larsen E., Bangsbøll S., Meinertz H., Carlsen E., Andersen A.N. Assisted reproduction in male cancer survivors: fertility treatment and outcome in 67 couples. Hum Reprod (2004); 19-12:2806-2810

33. Sieniawski M, Reineke T, Josting A, Nogova L, Behringer K, Halbsguth T, et al. Assessment of male fertility in patients with Hodgkin's lymphoma treated in the German Hodgkin Study Group (GHSG) clinical trials. Ann Oncol. 2008 Oct;19(10):1795-801. doi: 10.1093/annonc/mdn376.

34. Dickstein R.J., Shetty G., Meistrich M.L. Application of spermatogenesis suppression therapies for fertility preservation. In Fertility preservation in male cancer patients (2010);203-212 (80)

35. Thomson A.B., Wallace W.H.B., Sklar C. Testicular function. In Late Effects of Childhood Cancer (2004): 239-256.

36. Barri P.N., Veiga A., Boada M., Solé M. Embryo cryopreservation as a fertility preservation strategy. In Principles and Practice of Fertility Preservation (2011): 279-282.

37. Rodríguez J.M.T., Ávila L.B., Zúñiga I.C., Rodríguez F.G., Hernández V.M.V. Fertility and testicular function in a patient with germ cell extragonadal tumor. A report of a case. Ginecol Obstet Mex (2004) 75(1):46-49

38. Gunn HM, Rinne I, Emilsson H, Gabriel M, Maguire AM, Steinbeck KS. Primary Gonadal Insufficiency in Male and Female Childhood Cancer Survivors in a Long-Term Follow-Up Clinic. J Adolesc Young Adult Oncol. (2016) 5(4):344-350. doi: 10.1089/jayao.2016.0007

39. Thomas C, Cans C, Pelletier R, De Robertis C, Hazzouri M, Sele B, et al. No long-term increase in sperm aneuploidy rates after anticancer therapy: sperm fluorescence in situ hybridization analysis in 26 patients treated for testicular cancer or lymphoma. Clin Cancer Res. (2004) 10(19):6535-43. doi: 10.1158/1078-0432.CCR-04-0582.

40. Marmor D. Human fertility after antimitotic therapy. Bullet Cancer (1994) 81(9): 764-769

41. Thomas P.R.M., Mansfield M.D., Hendry W.F., Peckham M.J. The implications of scrotal interference for the preservation of spermatogenesis in the management of testicular tumours, Br J Sur (1997) 64(5): 352–354. Doi:10.1002/bjs.1800640515

42. Kovac V. Causes of fertile disturbances in oncological male patients. Radiology and Oncology (1996) 30(1): 46-54.

43. Bachaud J.M., Alzieu C., Bujan L. Effects of radiotherapy on adult testicular function. Andrologie (1995) 5(4): 476-485

44. Byrne J., Mulvihill J.J., Myers M.H., Connelly R.R., Naughton M.D., Krauss M.R. Effects of Treatment on Fertility in Long-Term Survivors of Childhood or Adolescent Cancer. New Eng J Med (1987) 317(21): 1315-1321.

45. Krause W., Pflüger K.H. Treatment with the Gonadotropin-Releasinghormone Agonist Buserelin to Protect Spermatogenesis against Cytotoxic Treatment in Young Men/Behandlung zum Schutz der Spermatogenese bei cytotoxischer Therapie mit dem Gonadotropin-Hormon Agonisten Buserelin. Andrologia (1989) 21(3): 265-270.

46. Naysmith T.E., Blake D.A., Harvey V.J., Johnson N.P. Do men undergoing sterilizing cancer treatments have a fertile future? Hum Reprod (1998) 13(11): 3250–3255, Doi:10.1093/humrep/13.11.3250

47. Asbjornsen G, Molne K, Klepp O, Aakvaag A. Testicular function after radiotherapy to inverted 'Y' field for malignant lymphoma. Scand J Haematol. (1976)17(2):96-100. doi: 10.1111/j.1600-0609.1976.tb01160.x

48. Kuber W., Lunglmayr G., Seitz W. Fertility in patients with testicular seminoma. Urologe - Ausgabe A (1980) 19(5): 272-275

49. Radford J.A., Clark S., Crowther D., Shalet S.M. Male fertility after VAPEC-B chemotherapy for Hodgkin's disease and non-Hodgkin's lymphoma. Br J Cancer. (1994) 69(2):379-81. doi: 10.1038/bjc.1994.69.

50. Meistrich M.L. Hormonal stimulation of the recovery of spermatogenesis following chemo- or radiotherapy. Review article. APMIS (1998)106(1):37-45; discussion 45-6. doi: 10.1111/j.1699-0463.1998.tb01317.x.

51. Lederlin P., Witz F., Aymard J.P. The influence of treatments of Hodgkin's disease upon male fertility. Ann Med Nancy (1978) 17(12): 1441-1448.

52. Carter M. Fertility in the testicular cancer patient. World J Urol. (1993)11(2):70-5. doi: 10.1007/BF00182032.

53. Yavetz H., Hauser R., Botchan A., Azem F., Yovel I., Lessing J.B et al. Pregnancy resulting from frozen-thawed embryos achieved by intracytoplasmic injection of cryopreserved sperm cells extracted from an orchidectomized, seminoma-bearing testis, causing obstructive azoospermia. Hum Reprod (1997) 12(12): 2836–2838. Doi: 10.1093/humrep/12.12.2836

54. Flamant F., Schweisguth O. Fertility and the progeny of children surviving cancer treatment. Bullet Cancer (1979); 66(2): 171-176

55. Pontonnier F., Plante P., Mansat A. Indications for the auto-conservation of sperm in urology. J Urol (1982) 88(2): 209-214

56. Jegou B., Velez De La Calle J.F. Spermatogenesis protection: Myth or reality? Contracept Fertil Sex (1993) 21(10): 744-748.

57. Slanina J., Musshoff K., Rahner T., Stiasny R., Barmeyer J., Baumeister L. Long-term side effects in irradiated patients with Hodgkin's disease. Int J Radiat Oncol Biol Phys. (1977) 2(1-2):1-19. doi: 10.1016/0360-3016(77)90003-7.

58. Gordon Jr. W., Siegmund K., Stanisic T.H., McKnight B., Harris I.T A study of reproductive function in patients with seminoma treated with radiotherapy and orchidectomy: (SWOG-8711). Southwest Oncology Group. Int J Radiat Oncol Biol Phys. (1997) 38(1):83-94. doi: 10.1016/s0360-3016(97)00235-6.

59. Kinsella T.J., Trivette G., Rowland J., Sorace R., Miller R., Fraass B., Steinberg S.M., Glatstein E., Sherins R.J. Long-term follow-up of testicular function following radiation therapy for early-stage Hodgkin's disease. J Clin Oncol (1989); 7(6): 718-724.

60. Hudson M.M., Greenwald C., Thompson E., Wilimas J., Marina N., Fairclough D., Kauffman W., Bozeman P., Mackert P.W., Abromowitch M., Jenkins J., Boulden T., Kun L. Efficacy and toxicity of multiagent chemotherapy and low-dose involved-field radiotherapy in children and adolescents with hodgkin's disease. J Clin Oncol (1996)11(1):100-108

61. Allen C., Keane D., Harrison R.F. A survey of Irish consultants regarding awareness of sperm freezing and assisted reproduction. Irish Medical Journal (2003) 96(1):23-25

62. Ghasemi B., Mehrjardi A.M., Jones C., Ghasemi N. Semen analysis of subfertility caused by testicular carcinoma. Int J Reprod Biomed. (2020) 18(7): 539–550.

63. Walter J.R., Lohman M.E., Kundu S.D., Xu S. A new fertility risk rating system for surgical, radiotherapy, and chemotherapy interventions used in testicular cancer. Translational Cancer Research (2016); 5: S778-S781.

64. Tinkanen H. Maintaining fertility in connection with cancer therapy. Duodecim; lääketieteellinen aikakauskirja (2011); 127(5): 480-485.

65. Yildirim B.A., Onal C. Radiotherapy in the management of testicular cancers. In Principles and Practice of Urooncology: Radiotherapy, Surgery and Systemic Therapy. (2017) 123-140.

66. Xi, Q., Zhu, L., Hu, J. et al. Successful pregnancy in a seminoma patient after fertility preservation. Chin. -Ger. J. Clin. Oncol. (2012) 11, 615–617. Doi: 10.1007/s10330-012-1006-9

67. Jahnukainen K., Ehmcke J., Hou M., Schlatt S. Testicular function and fertility preservation in male cancer patients. Best Pract Res Clin Endocrinol Metab. (2011) 25(2):287-302. doi: 10.1016/j.beem.2010.09.007.

68. Wang J., Muller C., Lin K. Optimizing fertility preservation for pre- and postpubertal males with cancer. Semin Reprod Med. (2013) 31(4):274-85. doi: 10.1055/s-0033-1345275.

69. Baird D.C., Meyers G.J., Hu J.S. Testicular Cancer: Diagnosis and Treatment. Am Fam Physician. (2018) 97(4):261-268.

70. Wood G.J.A., Hayden R.P., Tanrikut C. Successful sperm extraction and live birth after radiation, androgen deprivation and surgical castration for treatment of metastatic prostate cancer. Andrologia (2017) 49(1): e12578. Doi: 10.1111/and.12578

71. Marquis A., Kuehni C.E., Strippoli M.-P.F., Kühne T., Brazzola P. Sperm analysis of patients after successful treatment of childhood acute lymphoblastic leukemia with chemotherapy. Pediatr Blood Cancer (2010) 55(1):208-10

72. Nalesnik J.G., Sabanegh Jr. E.S., Eng T.Y., Buchholz T.A. Fertility in men after treatment for stage 1 and 2A seminoma. Am J Clin Oncol. (2004) 27(6):584-8. doi: 10.1097/01.coc.0000135736.18493.dd.

73. Buchler T., Freeman A., Harland S. Contralateral intratubular germ cell neoplasia in a patient with testicular cancer. Nat Clin Pract Urol (2008) 5(5):284-8.

74. Shin T., Miyata A., Arai G., Okada H. Fertility in testicular cancer patients. Jpn. J. Cancer Chemother (2015); 42(3): 267-271.

75. Mydlo J.H., Lebed B. Does brachytherapy of the prostate affect sperm quality and/or fertility in younger men? Scand J Urol Nephrol (2004) 38(3):221-4.

76. Dubey P., Wilson G., Mathur K.K., Hagemeister F.B., Fuller L.M., Ha C.S., et al. Recovery of sperm production following radiation therapy for Hodgkin's disease after induction chemotherapy with mitoxantrone, vincristine, vinblastine, and prednisone (NOVP). Int J Radiat Oncol Biol Phys.(2000) 46(3):609-17. doi: 10.1016/s0360-3016(99)00338-7.

77. Astafyeva L.I., Zhukov O.B., Kadashev B.A., Klochkova I.S., Kobyakov G.L., Poddubsky A.A., et al. Preservation of fertility in men with brain tumors. Russian Journal of Hum Reprod (2019) 25(1):74-82 Doi: 10.17116/repro20192501174

78. Huyghe E., Thonneau P.F., Plante P. Fertility after testicular cancer. Andrologie (2001); 11(4):221-225.

79. Putowski L., Kuczyński W. Strategies for fertility preservation after anti-cancer therapy. Ginekologia polska (2003) 74(8): 638-645.

80. Rives N., Verhaeghe F., Di Pizio P., Rives A. Fertility preservation. La Revue du praticien (2018); 68(2); 213-219.

81. Lass A., Akagbosu F., Brinsden P. Sperm banking and assisted reproduction treatment for couples following cancer treatment of the male partner. Hum Reprod Update (2008); 7(4): 370-377.

82. Brydøy M., Fosså S.D., Klepp O., Bremnes R.M., Wist E.A., Wentzel-Larsen T., Dahl O. Paternity following treatment for testicular cancer. J Natl Cancer Inst (2015); 97(21): 1580-1588.

83. Aisner J., Wiernik P.H., Pearl P. Pregnancy outcome in patients treated for Hodgkin's disease. J Clin Oncol. (1993)11(3):507-12. doi: 10.1200/JCO.1993.11.3.507.

84. Centola G.M., Keller J.W., Henzler M., Rubin P. Effect of Low-Dose Testicular Irradiation on Sperm Count and Fertility in Patients with Testicular Seminoma. J Androl (1994) 15(4): 608-610

85. Meistrich M.L., Wilson G., Mathur K., Fuller L.M., Rodriguez M.A., McLaughlin P et al. Rapid recovery of spermatogenesis after mitoxantrone, vincristine, vinblastine, and prednisone chemotherapy for Hodgkin's disease. J Clin Oncol (1992) 15(12): 3488-3495.

86. Jones T.H., Darne J.F. Self-administered subcutaneous human menopausal gonadotrophin for the stimulation of testicular growth and the initiation of spermatogenesis in hypogonadotrophic hypogonadism. Clin Endocrinol (Oxf). (1993) 38(2):203-8. doi: 10.1111/j.1365-2265.1993.tb00994.x

87. Petersen P.M., Skakkebæk N.E., Giwercman A. Gonadal function in men with testicular cancer: Biological and clinical aspects. APMIS (1998) 106(1): 24-36.

88. Corrado F., Feroldi P., Luraschi F., Bignardi M., Catalano G., Prina M. et al. Clinical dosimetry to evaluate the influence of peripheral dose on male fertility in radiotherapy. Physica Medica (1997);13(SUPPL 1): 97-99.

89. Fosså S.D., Åbyholm T., Normann N., Jetne V. Post-treatment Fertility in Patients with Testicular Cancer: III. Influence of Radiotherapy in Seminoma Patients. Br J Urol (1986);58: 315-319.

90. Hill M., Milan S., Cunningham D., Mansi J., Smith I., Catovsky D., Gore M., Zulian G., Selby P., Horwich A., O'Brien M., Nicolson M., Hickish T. Evaluation of the efficacy of the VEEP regimen in adult Hodgkin's disease with assessment of gonadal and cardiac toxicity. J Clin Oncol. (1995) 13(2):387-95. doi: 10.1200/JCO.1995.13.2.387.

91. Nallella K.P., Sharma R.K., Said T.M., Agarwal A. Inter-sample variability in post-thaw human spermatozoa. Cryobiology (2004) 49(2):195-9

92. 92. Schubert J., Held H.J., Kelly L.U., Tolkendorf E. Autologous frozen sperm--an alternative in infertility in the patient with invasively treated testicular tumor? Z Urol Nephrol (1989) 82(4): 209-216.

93. Bojanic N., Bumbasirevic U., Vukovic I., Bojanic G., Milojevic B., Nale D., et al Testis sparing surgery in the treatment of bilateral testicular germ cell tumors and solitary testicle tumors: A single institution experience. J Surg Oncol (2015)111(2):226-30

94. Fujita K., Tsujimura A. Fertility preservation for boys with cancer. Reprod Med Bio (2010); 9(4): 179-184.

95. El-Ahwany A., Samir H., Alahwany H. Using two different thawing temperatures and their effect on the motility recovery of human cryopreserved sperms in cancer patients. Middle East Fertil Soc J (2018) 23(4): 331-334.

96. Keene D.J.B., Sajjad Y., Makin G., Cervellione R.M. Sperm banking in the United Kingdom is feasible in patients 13 years old or older with cancer. J Urol (2012) 188(2); 594-597.

97. Arai Y., Kawakita M., Okada Y., Yoshida O. Sexuality and fertility in long-term survivors of testicular cancer. J Clin Oncol. (1997) 15(4):1444-8. doi: 10.1200/JCO.1997.15.4.1444

98. Schover L.R., Gonzales M., von Eschenbach A.C. Sexual and marital relationships after radiotherapy for seminoma. Urology (1986) 27(2):117-123.

99. Nijman J.M., Koops H.S., Oldhoff J., Kremer J., Jager S. Sexual function after bilateral retroperitoneal lymph node dissection for nonseminomatous testicular cancer. Arch Androl. (1987) 18(3):255-67. doi: 10.3109/01485018708988491

100. Krawczuk-Rybak M., Solarz E., Wojtkowska M., Wysocka J., Matysiak M., Gadomski A., et al. Gonadal function in young men after the treatment for Hodgkin lymphoma. Pediatr Endocrinol Diabetes Metab (2009)15(2):85-92

101. Li R.L., Zhang X.M. Advances in researches on inhibin B and male reproduction. Natl J Androl (2005); 11(4): 299-302

102. Schmiegelow M., Lassen S., Poulsen H.S., Schmiegelow K., Hertz H., Andersson A.-M., et al. Gonadal status in male survivors following childhood brain tumors. J Clin Endocrinol Metab (2001);86(6): 2446-2452

103. Stahl P.J., Stember D.S., Hsiao W., Schlegel P.N. Indications and strategies for fertility preservation in men. Clin Obstet Gynecol (2010); 53(4): 815-827.

104. De Luyk N., Pozzato G., Ricci G., Tamaro P., Manno M., Tomei F., Trombetta C. Pre-treatment and post-treatment fertility in young male patients affected by Hodgkin and non-Hodgkin lymphoma. Arch Ital Urol Androl (2012); 84(3):141-145.

105. Meseguer M., Molina N., García-Velasco J.A., Remohí J., Pellicer A., Garrido N. Sperm cryopreservation in oncological patients: A 14-year follow-up study. Fertil. Steril. (2006); 85(3): 640-645.

106. Van Casteren N.J., Boellaard W.P.A., Romijn J.C., Dohle G.R. Gonadal dysfunction in male cancer patients before cytotoxic treatment. Int. J. Androl. (2010); 33(1): 73-79

107. Ma L.H., Ding Q., Wang X. Advances in xenogeneic transplantation of spermatogonial stem cell and its bewilderment in clinical application. Natl. J. Androl. (2006)12(3):258-262.

108. David S., Orwig K.E. Spermatogonial Stem Cell Culture in Oncofertility. Urol Clin North Am (2020) 47(2):227-244

109. Lieng H., Chung P., Lam T., Warde P., Craig T. Testicular seminoma: Scattered radiation dose to the contralateral testis in the modern era. Pract Radiat Oncol (2018) 8(2): e57-e62

110. Haddad P., Karimi-Moghaddam Z., Esfahani M., Afkhami M., Farhan F., Amouzegar-Hashemi F. Thermoluminescence dosimetry of the dose received by scrotum and testes in radiotherapy of rectal cancer, compared to the point doses calculated by 3D-planning software. Phys Med. (2018) 45:143-145.

111. Vakalopoulos I., Dimou P., Anagnostou I., Zeginiadou T. Impact of cancer and cancer treatment on male fertility. Hormones (Athens). (2015) 14(4):579-89.

112. Schlatt S., Kliesch S. Male fertility protection. More than just sperm conservation? Gynakologische Endokrinologie (2012); 10(2):91-97.

113. De Lambert G., Poirot C., Guérin F., Brugières L., Martelli H. Preservation of fertility in children with cancer [La préservation de la fertilité dans les cancers de l'enfant]. Bull Cancer. (2015); 102(5):436-42.

114. Schouten N., Van Dalen T., Smakman N., Elias S.G., Van De Water C., Spermon R.J., Mulder L.S., Burgmans I.P.J. Male infertility after endoscopic Totally Extraperitoneal (Tep) hernia repair (Main): Rationale and design of a prospective observational cohort study. BMC Surg (2012); 12:7

115. Chiba K., Fujisawa M. Fertility preservation in men with cancer. Reprod Med Biol. (2014); 13(4): 177–184

116. Barak S. Fertility preservation in male patients with cancer. Best Pract Res Clin Obstet Gynaecol. (2019) 55:59-66

117. Ping P., Gu B.-H., Li P., Huang Y.-R., Li Z. Fertility outcome of patients with testicular tumor: Before and after treatment. Asian J Androl. (2014);16 (1): 107-111.

118. Howell S.J., Shalet S.M. Fertility preservation and management of gonadal failure associated with lymphoma therapy. Current oncology reports (2002); 4(5): 443-452

119. Mitchell R.T., Saunders P.T.K., Sharpe R.M., Kelnar C.J.H., Wallace W.H.B. Male fertility and strategies for fertility preservation following childhood cancer treatment. Endocr Dev (2009); 15:101-134

120. Magelssen H., Brydøy M., Fosså S.D. The effects of cancer and cancer treatments on male reproductive function. Nat Clinl Prac Urol (2006);3: 312–322.

121. Hamano I., Hatakeyama S., Ohyama C. Fertility preservation of patients with testicular cancer. Reprod. Med. Biol. (2017); 16(3): 240-251.

122. Radford J. Restoration of fertility after treatment for cancer. Horm Res. (2003); 59 Suppl 1:21-3.

123. Molnár Z., Berta E., Benyó M., Póka R., Kassai Z., Flaskó T., et al. Fertility of testicular cancer patients after anticancer treatment - Experience of 11 years. Pharmazie (2014); 69(6):437-441.

124. Thomson A.B., Critchley H.O.D., Kelnar C.J.H., Wallace W.H.B. Late reproductive sequelae following treatment of childhood cancer and options for fertility preservation. Best Pract Res Clin Endocrinol Metab. (2002);16(2):311-34.

125. Hennebicq S., des Cesos FF. Frequency and results of the use of cryopreserved semen. Andrologie (2004);14(4):398-403.

126. Schröder A.K., Diedrich K., Ludwig M. Strategies for preventing chemotherapy- and radiotherapy-induced gonadal damage. Am J Cancer (2004); 3(2): 97-117

127. Albers P. Germ-cell tumors and fertility. Reproduktionsmedizin (2000); 16(1):55-61.

128. Ginsberg J.P. Gonadotoxicity of cancer therapies in pediatric and reproductive-age males. In Oncofertility Medical Practice: Clinical Issues and Implementation (2012); 15-23

129. Ray K., Choudhuri R. Effects of Radiation on the Reproductive System. Reprod Dev Toxicol (2011); 291-299. Doi: 10.1016/B978-0-12-382032-7.10022-0

130. Kiserud C.E., Magelssen H., Fedorcsak P., Fosså S.D. Gonadal function after cancer treatment in adult men. Tidsskrift for den Norske Laegeforening (2008); 128(4):461-465 (

131. Brito V.N., Berger K., Mendonca B.B. Male hypogonadism: Childhood diagnosis and future therapies. Pediatric Health (2010); 4(5): 539-555. Doi: 10.2217/phe.10.50

132. Horne G, Atkinson A, Brison DR, Radford J, Yin JAL, Edi-Osagie ECO, et al. Achieving pregnancy against the odds: successful implantation of frozen–thawed embryos generated

by ICSI using spermatozoa banked prior to chemo/radiotherapy for Hodgkin's disease and acute leukaemia: Case Report. Hum Reprod (2001) 16(1): 107–109, Doi: 10.1093/humrep/16.1.107

133. Feneux D. Hematologic malignancies: Fertility in males and couples I. Fertility in males and couples [Hémopathies malignes: Sexualité, fertilité et grossesse I. Fertilité des couples et fertilité masculine]. Hematologie (2001); 7(2): 115-120.

134. Efthekar M, Mohammadin F, Yousefnejad F, Molaei B, Aflatoonian A. Comparison of conventional IVF versus ICSI in non-male factor, normoresponder patients. Int J Reprod Biomed. (2012) 10(2): 131-136.

135. ATabei T., Yoshida M., Oouchi H., Suwa Y. A case of metachronous bilateral testicular tumor treated by partial orchiectomy and local radiation (2013); 67-12:979-982.

136. Wyns, C. Cryopreservation and transplantation of testicular tissue. Principles and Practice of Fertility Preservation (2011): 209-224

137. Grinspon R.P., Arozarena M., Prada S., Bargman G., Sanzone M., Morales Bazurto M., et al. Safety of standardised treatments for haematologic malignancies as regards to testicular endocrine function in children and teenagers. Hum Reprod. (2019) 34(12):2480-2494. doi: 10.1093/humrep/dez216.

138. Celik-Ozenci C. Spermatogenesis and testicular function. In Fertility Preservation: Emerging Technologies and Clinical Applications (2012); 245-260. Doi: 10.1007/9781441917836_19

139. De Palma A, Vicari E, Palermo I, D'Agata R, Calogero AE. Effects of cancer and anti-neoplastic treatment on the human testicular function. J Endocrinol Invest. (2000) 23(10):690-6. doi: 10.1007/BF03343795.

140. Vermeulen M., Del Vento F., Kanbar M., Ruys S.P.D., Vertommen D., Poels J., et al. Generation of organized porcine testicular organoids in solubilized hydrogels from decellularized extracellular matrix. Int. J. Mol. Sci. (2019) 20(21), 5476

141. Vermeulen M., Poels J., de Michele F., des Rieux A., Wyns C. Restoring Fertility with Cryopreserved Prepubertal Testicular Tissue: Perspectives with Hydrogel Encapsulation, Nanotechnology, and Bioengineered Scaffolds. Ann Biomed Eng. (2017); 45(7):1770-1781.

142. Sönmezer M., Özkavukçu S. Fertility preservation in females with malignant disease-1: Causes, clinical needs and indications. Turk J Hematol (2009); 26(3): 106-113.

143. Riboldi M., Marqués Marí A.I., Simón C. Stem cells and fertility preservation in males. In Fertility Preservation: Emerging Technologies and Clinical Applications (2012) 345-352

144. Mohazzab A., Heidari M., Salehkhou S., Jeddi-Tehrani M., Akhondi M.M. Fertility preservation in men after cancer treatment; a review article. J Reprod Infert (2011) 11(2): 73-84.

145. De Felice F., Marchetti C., Marampon F., Cascialli G., Muzii L., Tombolini V. Radiation effects on male fertility. Andrology (2019) 7(1): 7-2

146. Barber H.R.K. The effect of cancer and its therapy upon fertility. Intl J Fertil (1981); 26(4):250-259.

147. Ohl D.A., Sonksen J. What are the chances of infertility and should sperm be banked? Semin Urol Oncol (1996); 14(1): 36-44.

148. Schubert J., Held H.J., Kelly L.U., Tolkendorf E. Autologous frozen sperm--an alternative in infertility in the patient with invasively treated testicular tumor? Z Urol Nephrol (1989);82(4):209-216.

149. Joos H., Sedlmayer F., Gomahr A., Rahim H.B.K., Frick J., Kogelnik H.D., Rettenbacher L. Endocrine profiles after radiotherapy in stage I seminoma: impact of two different radiation treatment modalities. Radiother Oncol. (1997) 43(2):159-62. doi: 10.1016/s0167-8140(97)00052-2.

150. Berthelsen J.G. Testicular cancer and fertility. Int J Androl (1987); 10(1 SPEC): 371-380.

151. Specht L., Geisler C., Hansen M.M., Skakkebæk N.E. Testicular function in young men in long-term remission after treatment for the early stages of Hodgkin's disease. Scand J Haematol (1984) 33(4): 356-362.

152. Petersen P.M., Giwercman A., Skakkebaek N.E., Rorth M. Gonadal function in men with testicular cancer. Semin Oncol (1998) 25(2): 224-234.

153. Bujan L., Mieusset R. Protection gonadique contre les effets des chimiothérapies et/ou radiothérapies chez l'homme: applications cliniques. Androl. 5, 504–511 (1995). Doi:10.1007/BF03034535

154. Barr R.D., Clark D.A., Booth J.D. Dyspermia in men with localized Hodgkin's disease. A potentially reversible, immune-mediated disorder. Med Hypotheses (1993); 40(3): 165-168.

155. DeSantis M., Albrecht W., Höltl W., Pont J. Impact of cytotoxic treatment on long-term fertility in patients with germ-cell cancer. Int J Cancer (1999) 83(6): 864-865

156. Chakraborti P.R., Neave F. Recovery of fertility 14 years following radiotherapy and chemotherapy for testicular tumour. Clin Oncol (1993) 5(4); 253-254.

157. Rustin G.J.S., Pektasides D., Bagshawe K.D., Newlands E.S., Begent R.H. Fertility after chemotherapy for male and female germ cell tumours. Int J Androl (1987)10 (1SPEC) 389-392.

158. Schover L.R. Sexuality and fertility in urologic cancer patients. Cancer (1987) 60 (3S): 553-558.

159. Christensen T.B., Daugaard G., Geertsen P.F., Von Der Maase H. Effect of chemotherapy on carcinoma in situ of the testis. Ann Oncol (1998) 9(6): 657-660.

160. Peckham M. Testicular cancer. Acta Oncologica (1988) 27(4): 439-453

161. Gruber G., Schwegler N. Low-dose testicular irradiation in seminoma patients. In-vivo dosimetry. Strahlenther Onkol (1999) 175(4); 185-189

162. Eghbali H, Papaxanthos-Roche A. The impact of lymphoma and treatment on male fertility. Expert Rev Hematol. (2010) 3(6):775-88. doi: 10.1586/ehm.10.70.

163. Hughes PD. Partial orchidectomy for malignancy with consideration of carcinoma in situ. ANZ J Surg. (2006)76(1-2):92-4. doi: 10.1111/j.1445-2197.2006.03660.x.

164. Ogle S.K., Hobbie W.L., Carlson C.A., Meadows A.T., Reilly M.M., Ginsberg J.P. Sperm banking for adolescents with cancer. J Pediatr Oncol Nurs. (2008) 25(2):97-101. doi: 10.1177/1043454207311922.

165. Herrmann T., Thiede G., Trott K.-R., Voigtmann L. Nachkommen präkonzeptionell bestrahlter Eltern. Abschlussbericht einer Longitudinalstudie 1976-1994 und Empfehlungen zur Patientenberatung [Offsprings of preconceptionally irradiated parents. Final report of a longitudinal study 1976-1994 and recommendations for patients' advisory]. Strahlenther Onkol. (2004)180(1):21-30. German. doi: 10.1007/s00066-004-1223-4.

166. Casbas J.M.G., Domínguez M.C. Demand and utilization of a sperm bank in oncological patients: Cryopreservation of pre-chemotherapy, pre-radiotherapy and pre-surgical semen. Arch Espan Urol (2004); 57(9): 1.017-1.02

167. González Casbas J.M., Calderay Domínguez M. Requests for utilization of a semen bank among oncological patients. Semen cryopreservation prior to chemotherapy, radiotherapy and surgery. Arch Españ Urol (2004); 57(9): 1017-1020

168. Rötgens J., Van Belle S. The preservation of male fertility before, during and after cancer treatment: Current state of affairs. Tijdschr Geneeskd (2015); 71-12:797-806

169. Marec-Berard P., Dubois C., Giscard D'Estaing S., Pacquement H., Brugières L., Laurence V, et al. An information booklet on the semen freezing intended for adolescents and young adults treated for cancer and the evaluation of its use in paediatric oncology centres in France: Preliminary results. Oncologie (2013) 15(5):255-259

170. Garolla A., Pizzol D., Bertoldo A., Ghezzi M., Carraro U., Ferlin A., Foresta C. Testicular cancer and HPV semen infection. Front Endocrinol (Lausanne). (2012) 3:172. doi: 10.3389/fendo.2012.00172.

171. Jing Y.-X., Zhang L.-L., Li H.-X., Yue F., Wang N., Xue S.-L., Wang Y.-Q., Zhang X.-H. Fertility preservation in cancer patients. Reprod Dev Med (2021); 5(1):44-54 Doi: 10.4103/2096-2924.309789

172. Borgmann-Staudt A., Sommerhäuser G., Balcerek M. Fertility preservation in children and adolescents with cancer [Fertilitätserhalt bei Tumoren im Kindes- und Jugendalter]. Onkologeco (2021);27(5): 441-446. Doi: 10.1007/s00761-021-00908-9

173. Cobo, A. Clinical outcome after oocyte cryopreservation for elective fertility preservation. In Preventing Age Related Fertility Loss (2017): 117-124.

174. Dittrich R., Binder H., Mueller A., Maltaris T., Hoffmann I., Oppelt P.G, et al. Gonadal toxicity. Options for fertility preservation for patients facing the loss of gonadal function. Gynakologe (2008) 41(8): 613-620. Doi: 10.1007/s00129-008-2174-0

175. Verhaeghe F., Rives N. Sperm conservation in 2016: Who and how? Progres en Urologie - FMC (2017) 27(1): F9-F13. Doi: 10.1016/j.fpurol.2016.09.002

176. Dieckmann KP, Claßen J, Souchon R, Loy V. Management of testicular intraepithelial neoplasia (TIN) - A review on the foundation of evidence based medicine (EBM) [Therapie der testikulären intraepithelialen neoplasie (TIN) - Eine übersicht auf grundlage der evidenzbasierten medizin (EBM)]. Wien Klin Wochenschr (2001);113(1-2):7-14.

177. Hempel D., Chrenowicz R., Filipowski T., Wojtukiewicz M.Z., Sierko E. Testicular dose contributed by X-ray volume image-(XVI)-guided intensity-modulated radiotherapy (IMRT) in prostate cancer patients. Nowotwory (2020); 70(2): 47-53 Doi: 10.5603/NJO.2020.0012

178. Brusamolino E., Lunghi F., Orlandi E., Astori C., Passamonti F., Barate C., et al. Treatment of early-stage Hodgkin's disease with four cycles of ABVD followed by adjuvant radiotherapy: Analysis of efficacy and long-term toxicity. Haematologica (2000); 85(10): 1032-1039 (142)

179. Klepfish A., Shvidel L., Shtalrid M., Haran M., Berrebi A. High rate of response and low rate of complications in hodgkin's disease treated with mopp-ABV hybrid chemotherapy regimen with limited radiotherapy for areas of bulky disease: 15 years experience in a single institution. Blood (2000); 96(11 Part II):240b-241b.

180. Mazur-Roszak M., Tomczak P., Litwiniuk M., Markowska J. Oncology and infertility: Selected issues. Part II. Preservation of the reproductive function. Wspolczesna Onkologia (2005); 9(2): 65-68.

181. Horne G., Atkinson A.D., Pease E.H.E., Logue J.P., Brison D.R., Lieberman B.A. Live birth with sperm cryopreserved for 21 years prior to cancer treatment: Case report. Hum Reprod. (2004); 19(6): 1448-1449.

182. Catanzaro M., Piva L., Torelli T., Biasoni D., Stagni S., Milani A., et al. Funcion sparing surgery in uro-oncology: germ cell tumors of the testis. Urologia (2012);79 Suppl 19:15-9

183. Yuksel M.B., Gumus B., Özbek E., Nese N. A unique case of bilateral synchronous testicular tumor with concomitant bilateral diffuse intratubular germ cell neoplasia: Testis sparing surgery and local radiotherapy. Cur. Urol. (2013); 6(3): 165-168.

184. Brougham M.F.H., Wallace W.H.B. Subfertility in children and young people treated for solid and haematological malignancies. Brit. J. Haem. (2005); 131(2): 143-155.

185. Okada K., Fujisawa M. Recovery of spermatogenesis following cancer treatment with cytotoxic chemotherapy and radiotherapy. World J. Mens Health (2019); 37(2): 166-174.

186. Rives N., Macé B. Cryopreservation of testicular tissue in boys: How can the boy's fertility be preserved? Andrologie (2004); 14(4): 404-411

187. Rabah D.M., Wahdan I.H., Merdawy A., Abourafe B., Arafa M.A. Oncologists' knowledge and practice towards sperm cryopreservation in Arabic communities. Journal of Cancer Survivorship (2010); 4(3): 279-283.

188. Rives N., Milazzo J.-P., Sibert L., Liard-Zmuda A., Travers A., Arkoun B., et al. Fertility preservation in males. Medecine Therapeutique Medecine de la Reproduction, Gynecologie et Endocrinologie (2012); 14(2); 86-93.

189. Li Y., Zhang J., Zhang H., Liu B., Wang G., Cao M., et al. Importance and safety of autologous sperm cryopreservation for fertility preservation in young male patients with cancer. Medicine (2020); 99(15): e19589

190. Thomas C., Rousseaux S., De Robertis C., Pelletier R., Sele B., Hennebicq S. Male fertility and chromosome aneuploidy in sperm cells after radiotherapy or chemotherapy in patients with lymphoma or testicular cancer. Andrologie (2003); 13(4): 403-411.

191. Steinsvik E.A., Fosså S.D., Lilleby W., Eilertsen K. Fertility issues in patients with prostate cancer. BJU Int. (2008); 102(7): 793-795.

192. Colpi G.M., Contalbi G.F., Nerva F., Sagone P., Piediferro G. Testicular function following chemo-radiotherapy. Eur J Obstet Gynecol Reprod Biol (2004); 113: S2-S6.

193. Brougham M.F.H., Kelnar C.J.H., Sharpe R.M., Wallace W.H.B. Male fertility following childhood cancer: Current concepts and future therapies. Asian J Androl (2003); 5(4): 325-337.

194. Ortega C., Tournaye H. Impact of radiotherapy and chemotherapy on the testis. In Fertility Preservation: Emerging Technologies and Clinical Applications (2012): 261-270.

195. Anderson K.H., Romao R.L.P. Testicular tumors in children and adolescents: Long-term endocrine and fertility issues. Trans. Androl. Urol. (2020); 9(5): 2393-2399.

196. Huleihel M., Lunenfeld E. Approaches and technologies in male fertility preservation. Int. J. Mol. Sc. (2020); 21(15): 1-19.

197. Patel B., Rossi B.V. Preserving fertility in young patients with lymphoma: An overview. Blood and Lymphatic Cancer: Targets and Therapy (2015); 5:1-15

198. Isachenko E., Isachenko V., Sanchez R., Katkov I.I., Kreienberg R. Cryopreservation of spermatozoa: Old routine and new perspectives. Principles and Practice of Fertility Preservation (2011): 176-198.

199. Korte E., Balcerek M., Borgmann-Staudt A. Fertility impairment and possibilities of fertility protection following childhood cancer. Gynakologische Praxis (2017); 41(4):623-630.

200. Korte E., Balcerek M., Borgmann-Staudt A. Fertility impairment and possibilities of fertility protection following childhood cancer. Padiatrische Praxis (2016); 87(1): 61068

201. Ståhl O., Eberhard J., Jepson K., Spano M., Cwikiel M., Cavallin-Ståhl E., et al. The impact of testicular carcinoma and its treatment on sperm DNA integrity. Cancer. 2004 Mar 15;100(6):1137-44. doi: 10.1002/cncr.20068.

202. Poirot C., Sitbon L., Fortin A., Berthaut I., Jaudi S., Anastacio A., et al. Fertility and cancer. Presse Medicale (2013); 42(11): 1513-1520

203. Lushbaugh C.C., Casarett G.W. The effects of gonadal irradiation in clinical radiation therapy: A review. Cancer (1976) 37(2S): 1111-1120.

204. Rendtorff R., Hohmann C., Reinmuth S., Müller A., Dittrich R., Beyer M., et al. Hormone and Sperm Analyses after Chemo- and Radiotherapy in Childhood and Adolescence. Klin Padiatr. (2010) 222(3):145-9. doi: 10.1055/s-0030-1249658.

205. Aslam I., Fishel S., Moore H., Dowell K., Thornton S. Fertility preservation of boys undergoing anti-cancer therapy: A review of the existing situation and prospects for the future. Hum Reprod (2000);15(10): 2154-2159.

206. Raison N., Warrington J., Alnajjar H.M., Muneer A., Ahmed K. The role of partial orchidectomy in the management of small testicular tumours: Fertility and endocrine function. Andrology (2020) 8(5):988-995.

207. Bruheim K, Svartberg J, Carlsen E, Dueland S, Haug E, Skovlund E, Tveit KM, Guren MG. Radiotherapy for rectal cancer is associated with reduced serum testosterone and increased FSH and LH. Int J Radiat Oncol Biol Phys. (2008) 70(3):722-7. doi: 10.1016/j.ijrobp.2007.10.043.

208. Gunn HM, Rinne I, Emilsson H, Gabriel M, Maguire AM, Steinbeck KS. Primary Gonadal Insufficiency in Male and Female Childhood Cancer Survivors in a Long-Term Follow-Up Clinic. J Adolesc Young Adult Oncol. (2016) 5(4):344-350. doi: 10.1089/jayao.2016.0007.

209. Sargos P., Ferretti L., Henriques de Figueiredo B., Cornelis F., Belhomme S., Dallaudière B., et al. Radiotherapy after testicular-sparing surgery for bilateral or monorchide testicular tumours: An innovative approach. Cancer Radiother (2013);17(4):317-22

210. Grant and Ramasamy, The pituitary gland and erectile dysfunction: Causes, investigation and management. In Erectile Dysfunction: Causes, Risk Factors and Management (2012): 129-143

211. Huddart RA, Norman A, Moynihan C, Horwich A, Parker C, Nicholls E, Dearnaley DP. Fertility, gonadal and sexual function in survivors of testicular cancer. Br J Cancer. (2005) 93(2):200-7. doi: 10.1038/sj.bjc.6602677.

212. Trabado S., Maione L., Brailly-Tabard S., Young J. Male acquired hypogonadotropic hypogonadism: Diagnosis and treatment. Ann Endocrinol (Paris) (2012) 73(2):141-6.

213. Brito V.N., Berger K., Mendonca B. Male hypogonadism: Childhood diagnosis and future therapies. Pediatric Health (2010) 4(5): 539-555.

214. van der Kaaij MA, Heutte N, Le Stang N, Raemaekers JM, Simons AH, Carde P, et al. European Organisation for Research and Treatment of Cancer: EORTC Lymphoma Group; Groupe d'Etude des Lymphomes de l'Adulte. Gonadal function in males after chemotherapy for early-stage Hodgkin's lymphoma treated in four subsequent trials by the European Organisation for Research and Treatment of Cancer: EORTC Lymphoma Group and the Groupe d'Etude des Lymphomes de l'Adulte. J Clin Oncol. (2007) 25(19):2825-32. doi: 10.1200/JCO.2006.10.2020.

215. Schlatt S, Nieschlag E. Keimzelltransplantation als Methode zur Fertilitätserhaltung bei onkologischen Patienten [Germ cell transplantation as a tool for fertility preservation of oncological patients]. Klin Padiatr. (2001) 213(4):250-4. German. doi: 10.1055/s-2001-16856.

216. Heidenreich A. Testis-preserving surgery in bilateral testicular germ cell tumours. Br J Urol (1997) 79(2): 253-257.

217. Grigor K.M., Donohue J.P. Reproductive aspects of testicular germ cell cancer: General discussion. Eur Urol (1993) 23(1): 177-181

218. Sedlmayer F., Joos H., Deutschmann H., Rahim H., Merz F., Dieter Kogelnik H. Long-term tumor control and fertility after limited paraaortic radiotherapy in Stage I seminoma. Strahlenther Onkol (1999) 175(7): 320-324.

219. Bokemeyer C., Schmoll H.-J., van Rhee J., Kuczyk M., Schuppert F., Poliwoda H. Long-term gonadal toxicity after therapy for Hodgkin's and non-Hodgkin's lymphoma. Ann. Hematol. (1994) 68(3): 105-110.

220. Pfitzer C., Chen C.M., Wessel T., Keil T., Sörgel A., Langer T., et al. Dynamics of fertility impairment in childhood brain tumour survivors. J Cancer Res Clin Oncol (2014) 140(10):1759-67

221. Ebert A.K., Bals-Pratsch M., Seifert B., Reutter H., Rösch W.H. Genital and reproductive function in males after functional reconstruction of the exstrophy-epispadias complex--long-term results. Urology. (2008) 72(3):566-9; discussion 569-70. doi: 10.1016/j.urology.2007.11.166.

222. Shah T.A., Keye Jr. W.R. Fertility: Tissue and cell banking overview. Clin Lab Med. (2005) 25(3):557-69.

223. Rousset-Jablonski C., Chevillon F., Dhedin N., Poirot C. Fertility preservation in adolescents and young adults with cancer. J Clin Oncol. (2010); 28(32):4831-41

224. Youssry M., Schöpper B., Schultze-Mosgau A., Von Otte S., Griesinger G., Diedrich K., et al. Ongoing twin pregnancy after transfer of vitrified oocyte injected with sperm recovered from cryopreserved testicular tissue. Middle East Fertil. Soc. J. (2007);12(3):213-215

225. Gholami M., Ahmadi S.A.Y., Abaszadeh A., Khaki A. Protective effects of melatonin and ghrelin on spermatogenesis: A narrative review of the literature. Int J Reprod Biomed (2017);15(5):265-272

226. Blackhall, F., Atkinson, A., Maaya, M, Ryder WDJ, Horne G, Brison DR, et al. Semen cryopreservation, utilisation and reproductive outcome in men treated for Hodgkin's disease. Br J Cancer (2002) 87, 381–384 .

227. Schmidt K.T., Andersen A.N., Loft A., Ernst E., Andersen C.Y. Cancer and infertility. Ugeskrift for Laeger (2012); 174(41): 2455: 2459.

228. Ji Y. Fertility preservation for adolescent survivors with malignant tumor. Tumor (2013); 33(10): 935-938.

229. Kaneva K., Erickson L., Rowell E., Badawy S.M. Fertility preservation education for pediatric hematology-oncology fellows, faculty and advanced practice providers: a pilot study. Pediatr Hematol Oncol. (2021); 24:1-6

230. Fallat M.E., Hutter J. Preservation of fertility in pediatric and adolescent patients with cancer. Pediatrics. (2008);121(5): e1461-9.

231. David S., Orwig K.E. Fertility preservation in cancer patients. Biol Mamm Sperm (2017): 315-341.

232. von Horn K., Depenbusch M., Schultze-Mosgau A., Neumann K., Griesinger G. Fertility preservation in oncology patients. Onkology (2017); 23(11): 943-950.

233. Levine, J. Fertility preservation in children and adolescents with cancer. Minerva Pediatrica (2011); 63(1):49-59.

234. Redig A.J., Brannigan R., Stryker S.J., Woodruff T.K., Jeruss J.S. Incorporating fertility preservation into the care of young oncology patients. Cancer (2011); 117(1):1-10

235. Di Pietro M.L., Teleman A.A. Cryopreservation of testicular tissue in pediatrics: Practical and ethical issues. J Matern-Fetal Neonatal Med (2013); 26(15): 1524-1527

236. Shin D., Lo K.C., Lipshultz L.I. Treatment options for the infertile male with cancer. J Natl Cancer Inst. Monographs (2005); 34:48-50.

237. Gul M., Hildorf S., Dong L., Thorup J., Hoffmann E.R., Jensen C.F.S., et al. Review of injection techniques for spermatogonial stem cell transplantation. Hum. Reprod. Update (2020); 26(3): 368-391.

238. Martin J.R., Patrizio P. Options for fertility preservation in pediatric populations undergoing cancer chemotherapy. Pediatric Endocrinol Rev (2009); 6(2); 306-314.

239. Vermeulen M., Giudice M.-G., Del Vento F., Wyns C. Role of stem cells in fertility preservation: Current insights. Stem Cells Cloning (2012); 12: 27-48.

240. Gurgan T., Salman C., Demirol A. Pregnancy and Assisted Reproduction Techniques in Men and Women after Cancer Treatment. Placenta (2008); 29(2): 152-159.

241. Beretta G. Iatrogenic infertility. In Clinical Management of Male Infertility (2015):145-152.

242. Bazeos A., Al-Shawaf T., Lower A., Wilson C., Gedis Grudzinskas J. Preservation of reproductive capacity of cancer patients. Reprod Technol (2000); 10(1): 42-49.

243. Oldenburg J., Fosså S.D. Long-term toxicity after therapy for testicular cancer with special focus on sexual disorders. Urologe - Ausgabe A (2004); 48(4): 372-376.

244. Wallace W.H.B. Oncofertility and preservation of reproductive capacity in children and young adults. Cancer (2011); 117(20): 2301-2310.

245. Castellotti D.S., Cambiaghi A.S. Fertility preservation for oncologic patients. Rev. Bras. Hematol. Hemoter. (2008); 30(5): 406-410.

246. de Lambert G., Poirot C., Guérin F., Brugières L., Martelli H. Preservation of future fertility in pediatric patients with cancer. J Visc Surg (2018); 155: S41-S46.

247. Ginsberg J.P. New advances in fertility preservation for pediatric cancer patients. Curr Opin Pediatr. (2011) 23(1):9-13. doi: 10.1097/MOP.0b013e3283420fb6.

248. Dittrich R., Lotz L., Hackl J., Nichols-Burns S., Hildebrandt T., Schneider H., Hoffmann I., Beckmann M.W.

249. Astaf'eva L.I., Zhukov O.B., Kadashev B.A., Klochkova I.S., Kobyakov G.L., Poddubskiy A.A., Kalinin P.L. Reproductive disorders and preservation of fertility in males with benign and malignant brain tumors. Zh. Vopr. Nejrokhir. Im. N.N. Burdenko (2019); 83(2): 59-65.

250. Kyono K. Fertility preservation. J Mamm. Ova Res. (2013); 30(3): 101-108.

251. Vallone R., Buonfantino C., Conforti A., De Rosa P., Cariati F., Picarelli S., et al. An update about oncofertility. Biochimica Clinica (2017); 41(4): 322-334.

252. Lockwood G. Oocyte cryopreservation: time to come in out of the cold... Women's Health Medicine (2006); 3(3): 128-129.

253. Puscheck E., Philip P.A., Jeyendran R.S. Male fertility preservation and cancer treatment. Cancer Treat Rev. 2004 Apr;30(2):173-80. doi: 10.1016/j.ctrv.2003.07.005.

254. Amirjannati N., Sadeghi M., Hosseini Jadda S.H., Ranjbar F., Kamali K., Akhondi M.A. Evaluation of semen quality in patients with malignancies referred for sperm banking before cancer treatment. Andrologia. (2011) 43(5):317-20. doi: 10.1111/j.1439-0272.2010.01077.x.

255. Pauli S.A., Berga S.L., Shang W., Session D.R. Current status of the approach to assisted reproduction. Pediatr Clin North Am. (2009) 56(3):467-88, Table of Contents. doi: 10.1016/j.pcl.2009.04.001.

256. Moss J.L., Choi A.W., Fitzgerald Keeter M.K., Brannigan R.E. Male adolescent fertility preservation. Fertil Steril. (2016) 105(2):267-73. doi: 10.1016/j.fertnstert.2015.12.002.

257. Grigg A. he impact of conventional and high-dose therapy for lymphoma on fertility. Clin Lymphoma. (2004)5(2):84-8. doi: 10.3816/clm.2004.n.013.

258. Guérin J.-F. Cryoconservation de tissu testiculaire chez le garçon prépubère: indications et faisabilité [Testicular tissue cryoconservation for prepubertal boy: indications and

feasibility]. Gynecol Obstet Fertil. (2005) 33(10):804-8. French. doi: 10.1016/j.gyobfe.2005.07.033.

259. Preface. Fertility Cryopreservation (2010); xi-xii. Doi: 10.1017/CBO9780511730207.001

260. Sirohi B., Rohatgi T.B., Lambertini M. Oncofertility and COVID-19-cancer does not wait. Ecancermedicalscience. (2020)14:ed101. doi: 10.3332/ecancer.2020.ed101.

261. Kim S.-Y., Kim S.K., Lee J.R., Woodruff T.K. oward precision medicine for preserving fertility in cancer patients: existing and emerging fertility preservation options for women. J Gynecol Oncol. (2016) 27(2):e22. doi: 10.3802/jgo.2016.27.e22.

262. Anderson R.A. Fertility preservation techniques: laboratory and clinical progress and current issues. Reproduction. (2008)136(6):667-9. doi: 10.1530/REP-08-0270.

263. Gunasheela D., Gunasheela S. Strategies for fertility preservation in young patients with cancer: a comprehensive approach. Indian J Surg Oncol. (2014) 5(1):17-29. doi: 10.1007/s13193-014-0291-x.

264. Leyvraz Recrosio C., Vaucher L., Primi M.-P. Fertility preservation and cancer in the male [Préservation de la fertilité masculine et cancer]. Revue Medicale Suisse (2012); 8(365): 2335-2339

265. Vázquez M.R., García M.G., Piñón M.L., Rodríguez M.C., Cancelo C.B., Mallo R.O.F., et al. Fertility preservation program in cancer patientes [Programa de preservación de la fertilidad en pacientes oncológicos]. Revista Iberoamericana de Fertilidad y Reproduccion Humana (2015);32(4): 35-44

266. Küçük M., Bolaman A.Z., Yavaşoğlu I., Kadiköylü G. Fertility-preserving treatment options in patients with malignant hematological diseases. Turk J Haematol. (2012) 29(3):207-16. doi: 10.5505/tjh.2012.72681.

267. Zhao H., Jin L., Li Y., Zhang C., Wang R., Li Y., et al. Oncofertility: What can we do from bench to bedside? Cancer Lett. (2019) 442:148-160. doi: 10.1016/j.canlet.2018.10.023.

268. Dittrich R., Maltaris T., Hoffmann I., Oppelt P.G., Beckmann M.W., Mueller A. Fertility preservation in cancer patients. Minerva Ginecologica (2010); 62(1): 63-80.

269. Klipstein S., Fallat M.E., Savelli S., Katz A.L., MacAuley R.C., Mercurio M.R., COMMITTEE ON BIOETHICS; SECTION ON HEMATOLOGY/ONCOLOGY; SECTION ON SURGERY. Fertility Preservation for Pediatric and Adolescent Patients With Cancer: Medical and Ethical Considerations. Pediatrics. (2020) 145(3):e20193994. doi: 10.1542/peds.2019-3994.

270. Alexandroni, H., Shoham, G., Levy-Toledano, R. et al. Fertility preservation from the point of view of hematopoietic cell transplant specialists—a worldwide-web-based survey analysis. Bone Marrow Transplant (2019). 54, 1747–1755 doi:10.1038/s41409-019-0519z

271. Kort J.D., Eisenberg M.L., Millheiser L.S., Westphal L.M. Fertility issues in cancer survivorship. CA Cancer J Clin. (2014) 64(2):118-34. doi: 10.3322/caac.21205.

272. Ronn R, Holzer HEG. Oncofertility in Canada: The Impact of Cancer on Fertility. Current Oncology. (2013) 20(4):338-344. Doi:10.3747/co.20.1358

273. Botha M.H., Kruger T.F. A review of the incidence and survival of childhood and adolescent cancer and the effects of treatment on future fertility and endocrine development. S. Afr. J. Obstet. Gynaecol. (2012); 18(2): 48-53

274. Wilkes S., Coulson S., Crosland A., Rubin G., Stewart J. Experience of fertility preservation among younger people diagnosed with cancer. Hum Fertil (Camb). (2010) 13(3):151-8. doi: 10.3109/14647273.2010.503359.

275. Maltaris T., Koelbl H., Seufert R., Kiesewetter F., Beckmann M.W., Mueller A., Dittrich R. Gonadal damage and options for fertility preservation in female and male cancer survivors. Asian J. Androl. (2006); 8(5): 515-533.

276. Muñoz M., Santaballa A., Seguí M.A., Beato C., de la Cruz S., Espinosa J., et al. SEOM Clinical Guideline of fertility preservation and reproduction in cancer patients. Clin Transl Oncol. (2016) 18(12):1229-1236. doi: 10.1007/s12094-016-1587-9.

277. Donnez J., Kim S.S. Principles and practice of fertility preservation. Principles and Practice of Fertility Preservation (2011); 1-549. Doi: 10.1017/CBO9780511921896

278. Biedka M., Kuźba-Kryszak T., Nowikiewicz T., Zyromska A. Fertility impairment in radiotherapy. Contemp Oncol (Pozn). (2016) 20(3):199-204. doi: 10.5114/wo.2016.57814.

279. Amzai G., Stojanovic A. Preservation of fertility and of reproduction ability in lymphoma patients. Maced. J. Med. Sci. (2013);6(2)

280. Tamás S., Róbert P. Onkofertilitás és kezelési lehetőségei. Irodalmi áttekintés [Oncofertility and therapeutic modalities. Survey of literature]. Orv Hetil. (2017)158(18):683-691. Hungarian. doi: 10.1556/650.2017.30730.

281. Ginsberg JP. Educational paper: The effect of cancer therapy on fertility, the assessment of fertility and fertility preservation options for pediatric patients. Eur J Pediatr. (2011) 170(6):703-708.

282. Hardy K., Wright C., Rice S., Tachataki M., Roberts R., Morgan D., Spanos S., Taylor D. Future developments in assisted reproduction in humans. Reproduction. (2002) 123(2):171-83. doi: 10.1530/rep.0.1230171.

283. Overbeek A., van den Berg M., Louwé L., Wendel E., ter Kuile M., Kaspers G., Stiggelbout A., van Dulmen-Den Broeder E., Hilders C. Practice, attitude and knowledge of Dutch paediatric oncologists regarding female fertility. Neth J Med (2014); 72(5): 264-270.

284. Vassilakopoulou M., Boostandoost E., Papaxoinis G., de La Motte Rouge T., Khayat D., Psyrri A. Anticancer treatment and fertility: Effect of therapeutic modalities on reproductive system and functions. Crit Rev Oncol Hematol. (2016) 97:328-334. Doi: 10.1016/j.critrevonc.2015.08.002

285. Brusamolino E., Bacigalupo A., Barosi G., Biti G., Gobbi P.G., Levis A., et al. Classical Hodgkin's lymphoma in adults: guidelines of the Italian Society of Hematology, the Italian Society of Experimental Hematology, and the Italian Group for Bone Marrow Transplantation on initial work-up, management, and follow-up. Haematologica. (2009);94(4):550-65. doi: 10.3324/haematol.2008.002451.

286. Xu R., Centola G.M., Tanrikut C. Genitourinary cancer patients have worse baseline semen parameters than healthy sperm bankers. Andrology (2019)7(4):449-453

287. Onofre J., Baert Y., Faes K., Goossens E. Cryopreservation of testicular tissue or testicular cell suspensions: A pivotal step in fertility preservation. Hum Reprod Update. (2016) 22(6): 744–761
